# Effect of new carbonyl cyanide aromatic hydrazones on biofilm inhibition against methicillin resistant *Staphylococcus aureus*†

**DOI:** 10.1039/d0ra03124k

**Published:** 2020-05-07

**Authors:** Xueer Lu, Ziwen Zhang, Yingying Xu, Jun Lu, Wenjian Tang, Jing Zhang

**Affiliations:** School of Medicine, Anhui University of Science and Technology Huainan 232001 China; Anhui Prevention and Treatment Center for Occupational Disease, Anhui No. 2 Provincial People's Hospital Hefei 230041 China hfzj2552@163.com; School of Pharmacy, Anhui Medical University Hefei 230032 China

## Abstract

Carbonyl cyanide *m*-chlorophenylhydrazone (CCCP), as a protonophore, in combination with antibiotics exhibited potentiating antibacterial activity. To improve CCCP's potency and toxicity, a series of aromatic hydrazones were synthesized and their antimicrobial activity was evaluated; amongst them, compounds 2e and 2j with a strong *para*-electron-withdrawing substituent (–NO_2_ and –CF_3_) at the phenyl ring had the lowest MICs against both *S. aureus* and methicillin resistant *Staphylococcus aureus* (1.56 and 1.56 μM, respectively). Some compounds in combination with antibiotics exhibited potentiate Gram-positive antibacterial activity; compound 2e was found to display unaided or synergistic efficacy against MRSA. In particular, when compound 2e is combined with ofloxacin, it has a good synergistic effect against MRSA. Moreover, electron microscopy revealed that compound 2e inhibits biofilm formation and effectively eradicates preformed biofilm. MTT assay showed that compound 2e displays as low toxicity as CCCP. Overall, our data showed that the aromatic hydrazone is a promising scaffold for anti-staphylococcal drug development.

## Abstract 2

2e and 2j with strong *p*-NO_2_ and *p*-CF_3_ at phenyl ring had the lowest MICs against *S. aureus* and MRSA. 2e displayed unaided or synergistic efficacy against MRSA, especially combined with ofloxacin. EM revealed that 2e destroys biofilms and cell membranes.

## Graphical abstract

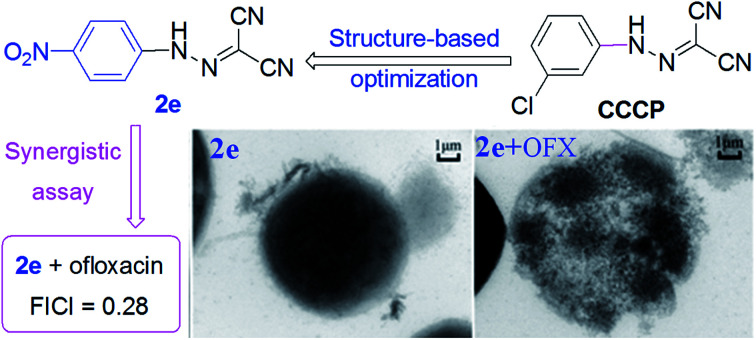

## 1. Introduction

Antimicrobial resistance (AMR) is an increasingly serious threat to global public health that requires a collaborative global approach across sectors. AMR largely reduces the antibiotic efficacies and increases health care costs, and the situation is getting worse due to the emergence of multidrug-resistant (MDR) bacterial pathogens, such as extended spectrum beta-lactamase *Enterobacteriaceae*, methicillin resistant *Staphylococcus aureus* (MRSA) and vancomycin-resistant enterococci (VRE).^1,2^ Therefore, there is now an urgent need to develop new antibacterial agents with novel targets and new approaches, which could be addressed by developing new antibacterial agents with unique chemical scaffolds.^3–5^

Mitochondria are well-known for their role as biosynthetic and bioenergetic organelles, which play a critical role in the innate immune response against viral and bacterial infections.^6,7^ As a chemical inhibitor of oxidative phosphorylation in the mitochondria, carbonyl cyanide *m*-chlorophenylhydrazone (CCCP) affects mitochondrial protein synthesis, causes an uncoupling of the proton gradient, acts essentially as an ionophore and reduces the ability of ATP synthase to function optimally. CCCP causes the gradual destruction of living cells and death of the organism by affecting the respiration and respiration-dependent phosphorylation.^8,9^ Antibiotic accumulation in Gram-negative bacteria is one of the major causes of AMR. CCCP was widely used to study cellular accumulation in Gram-negative bacteria for many small molecules^10–12^ due to its ability to collapse the proton motive force.^13^ As AMR spreads, a promising approach is to restore the effectiveness of existing drugs *via* co-administration with adjuvants that inhibit the growth of drug-sensitive pathogens.^4,14,15^ CCCP in combination with small molecules showed synergistic effect against most of the MDR pathogenic bacterial strains.^16–20^ CCCP in combination with antibiotics could potentiate antibacterial activity.

**Fig. 1:**
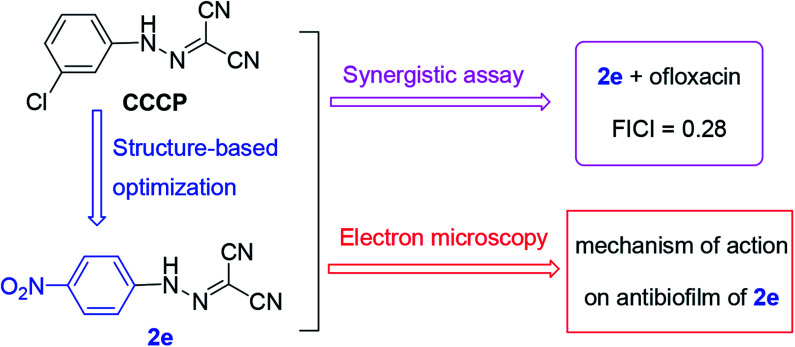
The general strategy in this work.

Since CCCP acts as a protonophore which disperses the membrane proton motive force by modifying the transmembrane electrochemical potential, it simultaneously causes toxicity to the cell of the host.^21,22^ Moreover, the concentration of synergistic antibacterial effect of CCCP is so high (at least 50 μM) that the effective dose may disrupt mitochondrial function to lead to toxicity.^23–25^ Therefore, in this work, a series of aromatic hydrazones were synthesized and evaluated for their antibacterial activity to try and improve antibacterial potency and reduce toxicity. The preliminary screening showed that aromatic hydrazones exhibited potential Gram-positive antibacterial activities. New compounds alone or in combination with antibiotics exhibited potentiate Gram-positive antibacterial activities. Therefore, the aromatic residue is a promising scaffold for further antibacterial modifications. Further, a plausible antibacterial mechanism was proposed and investigated *via* scanning electron microscopy (SEM) and transmission electron microscopy (TEM) (Fig. 1).

## 2. Results and discussion

### 2.1. Chemistry

**Scheme 1:**
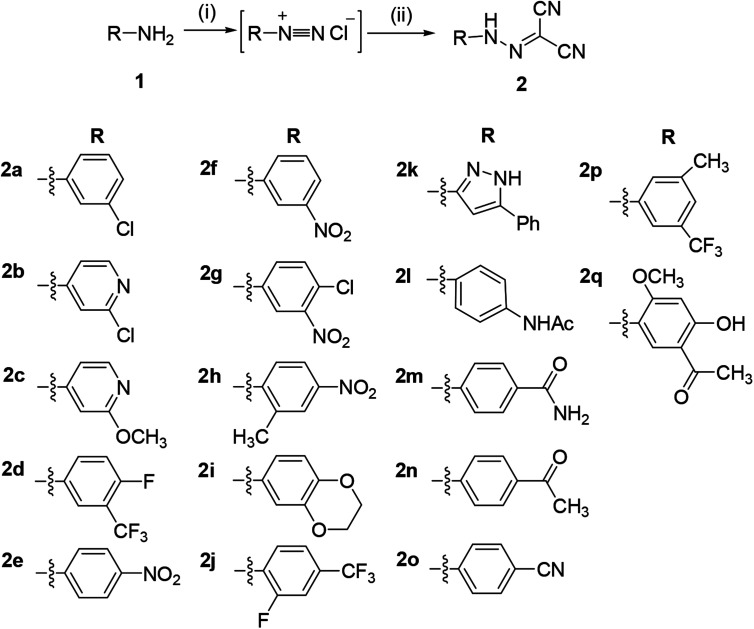
Synthesis of aromatic hydrazones 2a–2q. Reaction conditions and reagents: (i) HCl, NaNO_2_, 0 °C, 1 h; (ii) CH_2_(CN)_2_, CH_3_COONa, 0 °C, 2 h.

The synthetic route to compounds 2a–2q is illustrated in Scheme 1. The nitrosation of the aromatic amines (1) with nitrous acid (*in situ* from sodium nitrite and concentrated hydrochloric acid) led to aromatic diazonium salts, which can be used to next reaction without purification. The diazonium salt as the key intermediate underwent a condensation reaction with methylene of malononitrile to yield the title compound 2.

### 2.2. Antibacterial activity of compounds 2a–2q

**Fig. 2:**
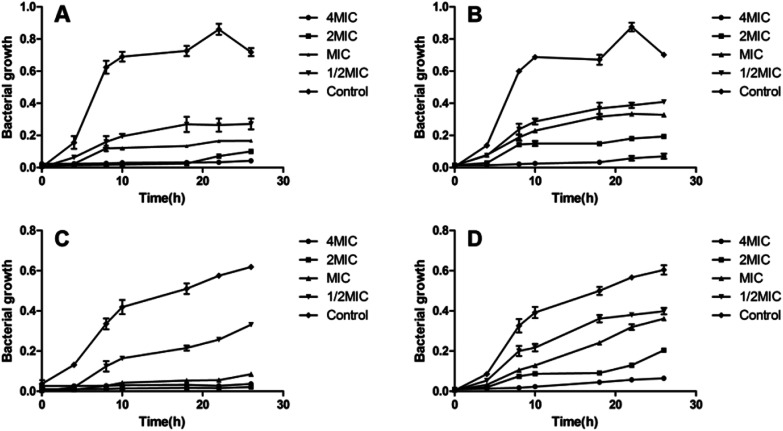
*S. aureus* and MRSA growth inhibition curves. Titration curves showing the effect of different concentrations of compounds 2e and 2j on the growth of *S. aureus* (A and B) and MRSA (C and D). Each OD point presented is the average values of three tests and all experiments are internally controlled. Data are presented as the mean ± standard deviation (*n* = 3).

In order to determine the antimicrobial potential of aromatic hydrazones, they were evaluated in either Mueller–Hinton (MH) broth or Sabouraud Dextrose Agar (SDA) using a micro-broth dilution method against a panel of bacteria and fungi, including two Gram-positive bacteria: *Staphylococcus aureus* ATCC 25923 (SA) and Methicillin Resistant *Staphylococcus aureus* (MRSA); two Gram-negative bacteria: *Pseudomonas aeruginosa* ATCC 9027 (PA) and *Escherichia coli* ATCC 8739 (EC); and *Candida albicans* ATCC 10231 (CA), respectively. The results showed that compounds 2a–2q showed no anti-microbial activity against Gram-negative bacteria and fungi (MICs > 200 μM, except for 2a, MIC = 50 μM for fungi), while some of compounds exhibited the moderate to high level of antibacterial activity against Gram-positive bacteria (Table 1). Amongst them, compounds 2e and 2j showed better activity than CCCP (2a) against both *S. aureus* and MRSA (MICs = 1.56 μM), which are even better than cefoxitin and linezolid, and similar with the MICs of ofloxacin. The growth inhibition effects of compounds 2a, 2e and 2j were further investigated against both *S. aureus* and MRSA. The results confirmed that both compounds 2e and 2j were able to inhibit the growth of *S. aureus* and MRSA effectively at the MIC or higher concentrations. Once the concentration drops down to half or less of the MICs, they could only slow down the growing rate of *S. aureus* and MRSA during logarithmic period, while the growth could be recovered after being incubated for longer time (Fig. 2).

**Table 1:** MIC (μM)^a^ of aromatic hydrazones against Gram-positive bacteria

Compounds^b^	SA	MRSA
2a	3.12	6.25
2b	>200	>200
2c	50	100
2d	100	100
2e	1.56	1.56
2f	25	50
2g	100	100
2h	6.25	12.50
2i	50	100
2j	1.56	1.56
2k	>200	>200
2l	200	>200
2m	200	>200
2n	25	100
2o	100	100
2p	50	100
2q	100	50
A	25	100
B	0.63	1.25
C	7.50	7.50

a MICs representing mean values of at least three replicates.

b A: cefoxitin, B: ofloxacin, C: linezolid.

### 2.3. SAR analysis

The structure–activity relationship (SAR) analysis are as followed: (i) when the aromatic ring of carbonyl cyanide *m*-chlorophenylhydrazone (CCCP) was substituted by heterocycle, the antibacterial activity against Gram-positive bacteria was significantly decreased (such as 2b, 2c and 2k); (ii) the phenyl ring with strong electron-withdrawing substituent (–NO_2_ and –CF_3_) showed the moderate antibacterial activity (such as 2d–2h, 2j and 2p); (iii) further, *para*-substituted group (–NO_2_ and –CF_3_) exhibited better activity than *meta*-substituted group, *e.g.* for *S. aureus* and MRSA (MIC values), 2e (1.56, 1.56 μM), 2h (6.25, 12.5 μM) > 2f (25, 100 μM), 2g (100, 100 μM); 2j (1.56, 1.56 μM) > 2d (100, 100 μM), 2p (50, 100 μM).

### 2.4. Cytotoxicity assays

**Fig. 3:**
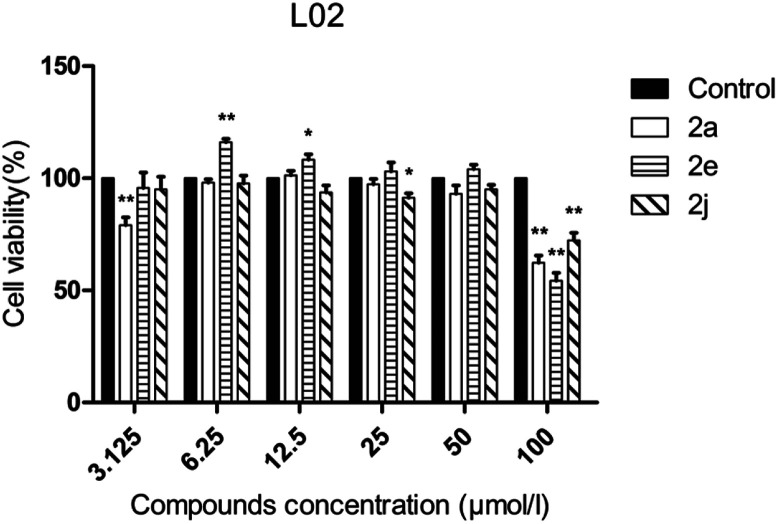
Cell viability assay of tested compounds to L02 cells. Data are presented as the mean ± standard error (*n* = 3), one-way ANOVA (*vs.* control), *P < 0.05, **P < 0.01.

The human hepatic L02 cells were treated with different concentrations of tested compound (3.125, 6.25, 12.5, 25, 50 and 100 μM), and cell viability was measured after 24 h using MTT method. As shown in Fig. 3, compounds 2a, 2e and 2j at the test concentrations (3.125–50 μM) had no obvious cytotoxicity against L02 cells, and the relative cell viabilities of treated cells were all more than 70%.

### 2.5. Checkerboard assay

To develop a feasible medical application, active compounds 2a, 2e and 2j were tested in combination with clinical antibiotics on SA and MRSA by checkerboard assay in order to evaluate their ability to improve the anti-bacterial activity.^20^ Each checkerboard test generates many different combinations and, by convention, the FIC value of the most effective combination was used in calculating the fractional inhibitory concentration index (FICI). FICI was calculated by adding both FICs:
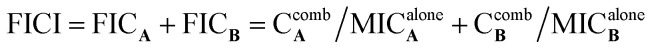
where 
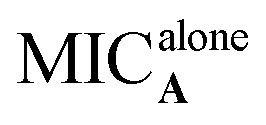
 and 
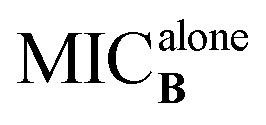
 are the MICs of compound A and B when acting alone and 
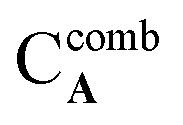
 and 
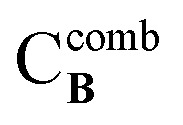
 are concentrations of compounds A and B at the isoeffective combinations. The FICI was interpreted as synergistic when it was ≤0.5, additional effects when 0.5 < FICI ≤ 1.0, indifferent when 1.0 < FICI ≤ 2.0, and antagonistic when FICI > 2.0, and any value between was interpreted as indifferent.

As shown in Table 2, when compound 2a was used in combination with antibiotics, MIC was reduced 2-fold, but for compound 2e, MIC was reduced 4-fold. Moreover, compounds 2a, 2e and 2j could significantly improve the performance of clinical antibiotics, for example, ofloxacin, cefoxitin and linezolid lowered their MIC values from 1.25, 50.0 and 7.5 μM to 0.04, 1.56 and 0.47 μM, respectively. Calculations of FIC and FICI (always less than 1.0) obtained by checkerboard assays on SA and MRSA showed at least additive effects of active compounds (2a, 2e and 2j) with clinical antibiotics. When compound 2e was combined with ofloxacin, FICI value of 0.28 suggested a synergistic effect. Therefore, worthy of note is the prophylactic purpose that low doses of clinical antibiotics plus a protonophore may be developed as an anti-MRSA therapy by inhibiting biofilm.

**Table 2:** Synergistic activity assays on MRSA

Compounds		MIC alone (μM)		MIC combination (μM)		FICI	Mode of action
A	B	A	B	A	B		
2a + ofloxacin		6.25	1.25	3.12	0.04	0.53	Additive
2e + ofloxacin		1.56	1.25	0.39	0.04	0.28	Synergy
2j + ofloxacin		1.56	1.25	0.78	0.04	0.53	Additive
2a + cefoxitin		6.25	50.00	3.12	1.56	0.53	Additive
2e + cefoxitin		1.56	50.00	0.78	1.56	0.53	Additive
2j + cefoxitin		1.56	50.00	0.78	1.56	0.53	Additive
2a + linezolid		6.25	7.50	3.12	0.47	0.56	Additive
2e + linezolid		1.56	7.50	0.78	0.47	0.56	Additive
2j + linezolid		1.56	7.50	0.78	1.90	0.75	Additive

### 2.6. Electron microscope

**Fig. 4:**
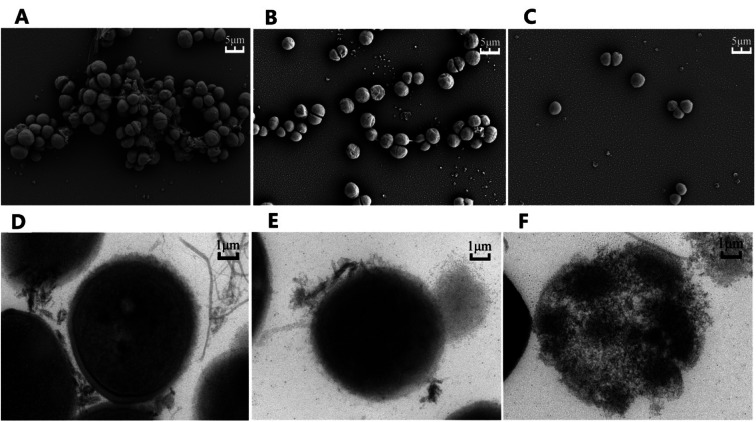
Electronic microscopies of MRSA (A–C). Scanning electronic microscopic images of MRSA and (D–F) Transmission electronic microscopic images of MRSA. (A and D) represent untreated bacteria. (B and E) represent bacteria treated with compound 2e at 1/2 MIC. (C and F) represent bacteria treated with compound 2e at 1/2 MIC and ofloxacin of 1/8 MIC.

To elucidate the effects of compound 2e on MRSA, both the Scanning Electron Microscopy (SEM) and Transmission Electron Microscopy (TEM) were used to observe the bacteria after being treated with either compound 2e alone or in combination with ofloxacin. As shown in Fig. 4, SEM results revealed that untreated MRSA form biofilms in normal growth condition, while the biofilms were eradicated when treated with compound 2e at 1/2 MIC concentration or in its combination with 1/8 MIC of ofloxacin. Furthermore, TEM results revealed that the regular cell conformation was destructed and the leakage of cellular substances under the treatment of compound 2e in combination with ofloxacin.

## 3. Conclusion

In summary, a series of aromatic hydrazones were synthesized and evaluated for their antibacterial activities. Some compounds showed potential antimicrobial activity against Gram-positive bacteria, amongst them, compounds 2e and 2j had the lowest MICs against both *S. aureus* and MRSA (1.56 μM), and the growth inhibition assay confirmed the inhibition effects. SAR showed that (i) for aromatic hydrazones containing heterocycles, the antibacterial activity against Gram-positive bacteria was significantly decreased (2a > 2b, 2c, 2k); (ii) the phenyl ring with strong electron-withdrawing substituent (–NO_2_ and –CF_3_) showed the moderate antibacterial activity (2d–2h, 2j and 2p); (iii) further, *para*-substituted group (–NO_2_ and –CF_3_) exhibited better activity than *meta*-substituted group, *e.g.* for *S. aureus* and MRSA (MIC values), 2e, 2h > 2f, 2g; 2j > 2d, 2p. Aromatic hydrazones in combination with clinical antibiotics exhibited better Gram-positive antibacterial activities, especially when compound 2e was used in combination with ofloxacin, in which the synergistic effect was observed. MTT assay showed that the toxicity of compound 2e was low as that of CCCP. Further, electron microscopy showed that compound 2e possesses the capability to inhibit the formation of biofilm and eradicate already existing biofilm. In sum, compound 2e displayed antibacterial activity against MRSA through inhibiting biofilm, especially improved the bactericidal effects of clinical antibiotics by synergistic effect. Therefore, the aromatic residue is a promising scaffold for further antibacterial modifications.

## 4. Materials and methods

### 4.1. Chemistry

All reagents were purchased from commercial sources and were used without further purification. Melting points (uncorrected) were determined on a XT4MP apparatus (Taike Corp., Beijing, China). ^1^H NMR and ^13^C NMR spectra were recorded on Bruker AV-600 or AV-400 MHz instruments in CDCl_3_. Chemical shifts are reported in parts per million (*δ*) downfield from the signal of tetramethylsilane (TMS) as internal standards. Coupling constants are reported in Hz. The multiplicity is defined by s (singlet), d (doublet), t (triplet), or m (multiplet). High resolution mass spectra (HRMS) were obtained on an Agilent 1260-6221 TOF mass spectrometry. Column and thin-layer chromatography (CC and TLC, resp.) were performed on silica gel (200–300 mesh) and silica gel GF_254_ (Qingdao Marine Chemical Factory) respectively.

### 4.2. General procedures for synthesis of 2-(2-arylhydrazono)malononitriles 2a–2q

To a solution of the aromatic amine (15 mmol) and concentrated HCl (37%, 13.8 mL) in H_2_O (75 mL) was dropwise added NaNO_2_ (15 mmol 1.04 g) in H_2_O (50 mL) for 1 h in an ice bath, and the mixture was stirred for 30 min. Then, the reaction solution was added to a solution of CH_2_(CN)_2_ (20 mmol, 1.26 mL) and NaOAc (31 mmol, 38.1 g) in H_2_O (130 mL) under continuous stirring at 0 °C. After 2 hours, the reaction mixture was filtrated, washed twice with water, and the residue was recrystallized from ethanol to give the title compounds 2a–2q.

#### *N*-(3-Chlorophenyl)carbonohydrazonoyl dicyanide (2a)

Yellow-green powder, yield, 80%; mp 131–133 °C; ^1^H NMR (400 MHz, DMSO-*d*_6_) *δ* 7.49–7.40 (m, 3H), 7.26 (d, *J* = 6.9 Hz, 1H). ^13^C NMR (151 MHz, CDCl_3_) *δ* 142.71, 135.01, 130.30, 125.54, 116.69, 114.73, 113.44, 109.53, 85.54.

#### *N*-(2-Chloropyridin-4-yl)carbonohydrazonoyl dicyanide (2b)

Yellow powder, yield, 83%; mp 92–94 °C; ^1^H NMR (400 MHz, DMSO-*d*_6_) *δ* 8.25 (d, *J* = 5.3 Hz, 1H), 7.29 (d, *J* = 5.1 Hz, 2H). ^13^C NMR (151 MHz, CDCl_3_) *δ* 153.60, 151.86, 149.81, 114.60, 111.33, 110.86, 110.60, 86.25. TOF-HRMS: *m*/*z* [M + H]^+^ calcd for C_8_H_4_ClN_5_: 206.0155; found: 206.0156.

#### *N*-(2-Methoxypyridin-4-yl)carbonohydrazonoyl dicyanide (2c)

Orange powder, yield, 78%; mp 42–43 °C; ^1^H NMR (400 MHz, CDCl_3_) *δ* 8.17 (d, *J* = 5.8 Hz, 1H), 6.90 (dd, *J* = 5.7, 1.7 Hz, 1H), 6.67 (s, 1H), 3.97 (s, 3H). ^13^C NMR (151 MHz, DMSO-*d*_6_) *δ* 163.06, 115.16, 110.59, 106.22 (2C), 95.33 (2C), 87.73, 54.98. TOF-HRMS: *m*/*z* [M + H]^+^ calcd for C_9_H_7_N_5_O: 202.0651; found: 202.0649.

#### *N*-(4-Fluoro-3-(trifluoromethyl)phenyl)carbonohydrazonoyl dicyanide (2d)

Earthy red powder, yield, 78%; mp 30 °C; ^1^H NMR (400 MHz, CDCl_3_) *δ* 10.06 (s, 1H), 7.82–7.42 (m, 2H), 7.31 (t, *J* = 9.1 Hz, 1H). ^13^C NMR (101 MHz, DMSO-*d*_6_) *δ* 157.75, 138.80, 122.89, 122.78, 119.27, 117.72, 115.36, 114.46, 110.15, 86.63.

#### *N*-(4-Nitrophenyl)carbonohydrazonoyl dicyanide (2e)

Bright yellow powder, yield, 81%; mp 40–41 °C; ^1^H NMR (400 MHz, DMSO-*d*_6_) *δ* 8.19 (d, *J* = 9.1 Hz, 2H), 7.49 (d, *J* = 9.1 Hz, 2H). ^13^C NMR (101 MHz, DMSO-*d*_6_) *δ* 142.71, 125.19 (2C), 118.14 (2C), 117.98, 113.20, 94.76, 82.41. TOF-HRMS: *m*/*z* [M + H]^+^ calcd for C_9_H_6_N_5_O_2_: 230.0547; found: 230.0550.

#### *N*-(3-Nitrophenyl)carbonohydrazonoyl dicyanide (2f)

Yellow powder, yield, 82%; mp 144–145 °C; ^1^H NMR (400 MHz, DMSO-*d*_6_) *δ* 8.21 (t, *J* = 2.1 Hz, 1H), 8.08–7.96 (m, 1H), 7.91–7.77 (m, 1H), 7.68 (t, *J* = 8.2 Hz, 1H). ^13^C NMR (151 MHz, DMSO-*d*_6_) *δ* 148.91, 144.78, 131.33, 123.26, 119.80, 115.30, 111.59, 110.85, 85.83.

#### *N*-(4-Chloro-3-nitrophenyl)carbonohydrazonoyl dicyanide (2g)

Yellow powder, yield, 80%; mp 40–41 °C; ^1^H NMR (400 MHz, DMSO-*d*_6_) *δ* 7.73 (s, 1H), 7.56 (d, *J* = 8.7 Hz, 1H), 7.48 (d, *J* = 8.7 Hz, 1H). ^13^C NMR (101 MHz, DMSO-*d*_6_) *δ* 158.90, 153.42, 136.88, 129.41, 126.08, 122.79, 121.01, 119.43, 81.46.

#### *N*-(2-Methyl-4-nitrophenyl)carbonohydrazonoyl dicyanide (2h)

Dark green powder, yield, 85%; mp 41 °C; ^1^H NMR (400 MHz, CDCl_3_) *δ* 9.57 (s, 1H), 8.18 (dt, *J* = 27.6, 12.5 Hz, 2H), 7.70 (d, *J* = 8.9 Hz, 1H), 2.51 (s, 3H). ^13^C NMR (101 MHz, CDCl_3_) *δ* 146.56, 144.00, 129.46, 126.58, 122.61, 118.70, 114.81, 110.23, 87.24, 17.47.

#### *N*-(2,3-Dihydrobenzo[*b*][1,4]dioxin-6-yl)carbonohydrazonoyl dicyanide (2i)

Earth orange powder, yield, 84%; mp 90 °C; ^1^H NMR (400 MHz, DMSO-*d*_6_) *δ* 6.99–6.94 (m, 1H), 6.92–6.87 (m, 1H), 4.25 (s, 4H). ^13^C NMR (101 MHz, DMSO-*d*_6_) *δ* 144.21, 142.25, 136.12, 118.20, 115.24, 110.81, 110.17, 105.94, 83.35, 64.68, 64.49.

#### *N*-(2-Fluoro-5-(trifluoromethyl)phenyl)carbonohydrazonoyl dicyanide (2j)

Orange powder, yield, 90%; mp 33–35 °C; ^1^H NMR (400 MHz, DMSO-*d*_6_) *δ* 7.80 (d, *J* = 6.9 Hz, 1H), 7.68–7.51 (m, 2H). ^13^C NMR (101 MHz, DMSO-*d*_6_) *δ* 155.03, 132.74, 126.42, 124.09, 124.06, 118.49, 117.50, 115.37, 110.80, 87.05. TOF-HRMS: *m*/*z* [M + Na]^+^ calcd for C_10_H_4_F_4_N_4_Na: 279.0264; found: 279.0261.

#### *N*-(5-Phenyl-1*H*-pyrazol-3-yl)carbonohydrazonoyl dicyanide (2k)

Yellow powder, yield, 89%; mp 138–139 °C; ^1^H NMR (400 MHz, DMSO-*d*_6_) *δ* 9.33 (s, 2H), 8.17 (d, *J* = 7.5 Hz, 2H), 7.66 (s, 1H), 7.53 (dt, *J* = 13.8, 7.1 Hz, 3H). ^13^C NMR (101 MHz, DMSO-*d*_6_) *δ* 156.98, 150.06, 143.24, 131.62, 130.41, 129.41 (2C), 127.14 (2C), 116.37, 105.62, 96.13. TOF-HRMS: *m*/*z* [M + H]^+^ calcd for C_12_H_8_N_6_: 237.0801; found: 237.0805.

#### *N*-(4-Acetamidophenyl)carbonohydrazonoyl dicyanide (2l)

Yellow powder, yield, 70%; mp 45–46 °C; ^1^H NMR (400 MHz, DMSO-*d*_6_) *δ* 10.07 (s, 1H), 7.63 (d, *J* = 8.8 Hz, 2H), 7.40 (d, *J* = 8.8 Hz, 2H), 2.05 (s, 3H). ^13^C NMR (101 MHz, DMSO-*d*_6_) *δ* 168.77, 137.82, 137.56, 120.18 (2C), 117.56 (2C), 115.42, 110.94, 83.40, 24.43.

#### *N*-(4-Carbamoylphenyl)carbonohydrazonoyl dicyanide (2m)

Dark yellow green powder, yield, 82%; mp 155–157 °C; ^1^H NMR (400 MHz, DMSO-*d*_6_) *δ* 7.96 (s, 1H), 7.90 (d, *J* = 8.8 Hz, 2H), 7.46 (d, *J* = 8.8 Hz, 2H), 7.33 (s, 1H). ^13^C NMR (101 MHz, DMSO-*d*_6_) *δ* 167.69, 146.72, 131.09 (2C), 129.38 (2C), 116.90, 116.29, 111.71, 84.09.

#### *N*-(4-Acetylphenyl)carbonohydrazonoyl dicyanide (2n)

Bright yellow powder, yield, 91%; mp 254–255 °C; ^1^H NMR (400 MHz, DMSO-*d*_6_) *δ* 7.93 (d, *J* = 8.6 Hz, 1H), 7.46 (d, *J* = 8.6 Hz, 1H), 2.51 (s, 1H). ^13^C NMR (101 MHz, DMSO-*d*_6_) *δ* 197.03, 150.90, 133.24, 130.23 (2C), 117.73 (2C), 113.00, 99.98, 82.71, 26.99.

#### *N*-(4-Cyanophenyl)carbonohydrazonoyl dicyanide (2o)

Yellow-green powder, yield, 88%; mp 116–117 °C; ^1^H NMR (400 MHz, DMSO-*d*_6_) *δ* 7.78 (d, *J* = 8.4 Hz, 2H), 7.51 (d, *J* = 8.4 Hz, 2H). ^13^C NMR (101 MHz, DMSO-*d*_6_) *δ* 149.82, 134.03 (2C), 119.57, 118.29 (2C), 116.88, 112.26, 106.62, 84.34.

#### *N*-(3,5-Bis(trifluoromethyl)phenyl)carbonohydrazonoyl dicyanide (2p)

Light yellow powder, yield, 70%; mp 113–115 °C; ^1^H NMR (400 MHz, CDCl_3_) *δ* 7.86 (s, 2H), 7.73 (s, 1H).

#### *N*-(5-Acetyl-4-hydroxy-2-methoxyphenyl)carbonohydrazonoyl dicyanide (2q)

Dark green powder, yield, 70%; mp 132–134 °C; ^1^H NMR (400 MHz, DMSO-*d*_6_) *δ* 12.58 (s, 1H), 7.79 (s, 1H), 6.69 (s, 1H), 3.95 (s, 3H), 2.59 (s, 3H). ^13^C NMR (101 MHz, DMSO-*d*_6_) *δ* 203.23, 162.86, 156.87, 123.39, 122.67, 114.81, 113.71, 110.20, 100.94, 85.12, 57.21, 27.67.

### 4.3. Culture conditions and treatments

L02 (normal human liver) cell lines were purchased from the Russian Cell Culture Collection (Institute of Cytology Russian Academy of Science, Saint Petersburg, Russia). L02 cells were maintained in Dulbecco's modified Eagle's medium (DMEM) (Invitrogen, USA) supplemented with 2 mM l-glutamine (Sigma-Aldrich, UK), 10% fetal bovine serum (Invitrogen, USA), 50 μg mL^−1^ gentamicin sulfate (Invitrogen, USA) at 37 °C and 5% CO_2_. All compounds were dissolved in 100% DMSO (Sigma-Aldrich, UK) to 100 mM stock solutions and diluted in completed DMEM immediately before addition to the assay plates. DMSO was maintained at a final concentration of 0.1%.

### 4.4. Minimum inhibitory concentrations (MICs)

The MICs of tested compounds were determined using Mueller–Hinton (MH) broth micro-broth dilution assay established by the Clinical Laboratory Standards Institute (CLSI) in 96-well micro-test plates. The final test concentration ranged from 0.39 to 200 μM and the bacterial inocula was 10^8^ CFU mL^−1^. After 18–20 hours of incubation at 37 °C, the MICs were determined to be the lowest concentration of tested compound that inhibited the apparent increase in microorganisms. Each experiment was repeated at least 3 times to report the MIC value.^26^

### 4.5. Inhibition of bacterial growth

The effect of concentrations ranging from 0.5 to 4 times MIC of the active compounds on the growth of *S. aureus* or MRSA was quantified after incubation at 35 °C for 0, 4, 8, 10, 18, 22 and 26 hours. At each time point, an aliquot (100 μL) was pipetted and measured for the *A*_450_ nm. The experiment was performed in three biologically independent assays, each tested in triplicate.

### 4.6. Checkerboard assays

The synergistic effect of the combination of clinical antibacterials with the tested compounds was determined by checkerboard microdilution assays. In brief, checkerboards were set up with double dilutions of compounds 2a (0–12.5 μM) or 2e (0–3.12 μM) or 2j (0–3.12 μM) in the horizontal wells and ofloxacin (0–2.5 μM) or cefoxitin (0–100 μM) or linezolid (0–14 μM) in the vertical wells. Then 50 μL each was arranged on the rows and columns of the plate, and 100 μL of MRSA was added to the wells and bacteria inocula of 5 × 10^8^ CFU mL^−1^. After incubation at 35 °C for 20 hours in 96-well micro-test plates. Aromatic hydrazones were further tested to determine their nature of interaction (synergy, antagonism, additive or no interaction) with ofloxacin, cefoxitin and linezolid and expressed as the fractional inhibitory concentration index (FICI) for each agent.

### 4.7. Cell viability

Cell viability was performed against L02 (normal human liver cell line) cells using the MTT assay. L02 cells were grown in DMEM containing 10% fetal calf serum, 100 units per mL penicillin and 100 μg mL^−1^ streptomycin at 37 °C in a 5% CO_2_ incubator. L02 cells were seeded at 1 × 10^4^ cells per well in 96-well micro-test plates. After 24 h of culture, the cells were treated with different concentrations of tested compound. After 24 h, 20 μL of 0.5 mg mL^−1^ MTT reagent was added to the cells and incubated for 4 h. After 4 h, the liquid in the well was discarded, and then 150 μL of DMSO was added to dissolve the formazan. The absorbance value (OD_570_) was measured at 570 nm. The cell percentage survival rate was calculated by setting the density of formazan formed in the blank group to 100% viability as a control. Cell viability (%) = compound (OD_570_)/blank (OD_570_) × 100%. Each compound was tested in triplicate.

### 4.8. Electron microscope

MRSA (ATCC 43300) was grown overnight at 37 °C on Mueller–Hinton Agar. The bacteria were harvested and the OD of bacteria suspended in MHB was adjusted to ∼0.5 MacFarlane units so as to give 5 × 10^7^ CFU mL^−1^. Bacteria were then aliquoted into 10 mL tubes and compound 2e dissolved in DMSO was added to give a final concentration ranging from 0.5 to 4 mg L^−1^ (two fold serial dilutions). After incubation at 37 °C for 24 h, the bacteria were harvested by centrifugation at 4000 rpm, and cell pellets were then re-suspended with 10 mM PBS, pH 7.2 and harvested at 4000 rpm. The bacteria were fixed using 2.5% glutaraldehyde for 3 h, following by washing with 0.1 M PBS (pH 7.2) for three times. The washing buffer was then removed and the bacteria were post-fixed in 1% OsO_4_ for 2 h. The OsO_4_ were then pipetted out into an osmium waste bottle and the bacteria were washed in PBS (pH 7.2) for three times. Fixed microbial pellets were processed in graded alcohols, propylene oxide, and araldite and cured for 48 h at 60 °C. Sample were finally stained with uranyl acetate and lead citrate before examine with Hitachi TEM system at an accelerating voltage of 80 kV. The SEM model used is the Hitachi su8100 at 3.0 kV voltage.^27^

### 4.9. Statistical analysis

All results were expressed as mean values ± standard deviation. One-way analysis of variance followed by Dunnett's post hoc test was used for all comparisons.

## Conflicts of interest

There are no conflicts of interest to declare.

## Supplementary Material

RA-010-D0RA03124K-s001

## References

[cit1] O'Connell K. M. G., Hodgkinson J. T., Sore H. F., Welch M., Salmond G. P. C., Spring D. R. (2013). Angew. Chem., Int. Ed..

[cit2] Tommasi R., Brown D. G., Walkup G. K., Manchester J. I., Miller A. A. (2015). Nat. Rev. Drug Discovery.

[cit3] Wright G. D. (2017). Nat. Prod. Rep..

[cit4] Tyers M., Wright G. D. (2019). Nat. Rev. Microbiol..

[cit5] Cattoir V., Felden B. (2019). J. Infect. Dis..

[cit6] Weinberg S. E., Sena L. A., Chandel N. S. (2015). Immunity.

[cit7] Richter M. F., Drown B. S., Riley A. P., Garcia A., Shirai T., Svec R. L., Hergenrother P. J. (2017). Nature.

[cit8] Cavari B. Z., Avi-Dor Y. (1967). Biochem. J..

[cit9] Mishra P., Chan D. C. (2016). J. Cell Biol..

[cit10] Lomovskaya O., Warren M. S., Lee A., Galazzo J., Fronko R., Lee M., Blais J., Cho D., Chamberland S., Renau T., Leger R., Hecker S., Watkins W., Hoshino K., Ishida H., Lee V. J. (2001). Antimicrob. Agents Chemother..

[cit11] Davis T. D., Gerry C. J., Tan D. S. (2014). ACS Chem. Biol..

[cit12] Cai H., Rose K., Liang L.-H., Dunham S., Stover C. (2009). Anal. Biochem..

[cit13] Ghoul M., Pommepuy M., Moillo-Batt A., Cormier M. (1989). Appl. Environ. Microbiol..

[cit14] Michel J. B., Yeh P. J., Chait R., Moellering Jr R. C., Kishony R. (2008). Proc. Natl. Acad. Sci. U. S. A..

[cit15] Allen R. C., Brown S. P. (2019). mBio.

[cit16] Tharmalingam N., Jayamani E., Rajamuthiah R., Castillo D., Fuchs B. B., Kelso M. J., Mylonakis E. (2017). Future Med. Chem..

[cit17] Stokes J. M., Macnair C. R., Ilyas B., French S., Côté J.-P., Bouwman C., Farha M. A., Sieron A. O., Whitfield C., Coombes B. K., Brown E. D. (2017). Nat. Microbiol..

[cit18] Sinha D., Pandey S., Singh R., Tiwari V., Sad K., Tandon V. (2017). Sci. Rep..

[cit19] Chen C., Yang K. (2019). Bioorg. Chem..

[cit20] Sabatini S., Piccioni M., Felicetti T., De Marco S., Manfroni G., Pagiotti R., Nocchetti M., Cecchetti V., Pietrella D. (2017). RSC Adv..

[cit21] Park J. W., Lee S. Y., Yang J. Y., Rho H. W., Park B. H., Lim S. N., Kim J. S., Kim H. R. (1997). Biochim. Biophys. Acta.

[cit22] Mahamoud A., Chevalier J., Alibert-Franco S., Kern W. V., Pagès J. M. (2007). J. Antimicrob. Chemother..

[cit23] Kansaku K., Takeo S., Itami N., Kin A., Shirasuna K., Kuwayama T., Iwata H. (2017). PLoS One.

[cit24] Zhang X., Zhang Y., Wang F., Wang C., Chen L., Liu H., Lu H., Wen H., Zhou T. (2018). Int. J. Antimicrob. Agents.

[cit25] Fedorowicz J., Sączewski J., Konopacka A., Waleron K., Lejnowski D., Ciura K., Tomašič T., Skok Ž., Savijoki K., Morawska M., Gilbert-Girard S., Fallarero A. (2019). Eur. J. Med. Chem..

[cit26] Wiegand I., Hilpert K., Hancock R. E. (2008). Nat. Protoc..

[cit27] Xu K., He S., Chen S., Qiu G., Shi J., Liu X., Wu X., Zhang J., Tang W. (2018). Eur. J. Med. Chem..

