# Supplementary material for: Effect of new carbonyl cyanide aromatic hydrazones on biofilm inhibition against methicillin resistant Staphylococcus aureus
Source: RSC Adv. 2020 May 7;10(30):17854–61. doi: 10.1039/d0ra03124k (PMC9053579; doi:10.1039/d0ra03124k)
Supplement: RA-010-D0RA03124K-s001 [file RA-010-D0RA03124K-s001.pdf]

Electronic Supplementary Material (ESI) for RSC Advances.
This journal is © The Royal Society of Chemistry 2020

Effect of new carbonyl cyanide aromatic hydrazones on biofilm inhibition against Methicillin Resistant *Staphylococcus Aureus*

Xueer Lu,[a,b] Ziwen Zhang,[c] Yingying Xu,[c] Jun Lu,[a] Wenjian Tang,[c] Jing Zhang [a,b,*]

[a] *School of Medicine, Anhui University of Science and Technology, Huainan 232001, China*

[b] *Anhui Prevention and Treatment Center for Occupational Disease, Anhui No. 2 Provincial People's Hospital, Hefei 230022, China*

[c] *School of Pharmacy, Anhui Medical University, Hefei 230032, China*

Content

Copies of NMR spectra of compounds 2a-2q **S2–S18**

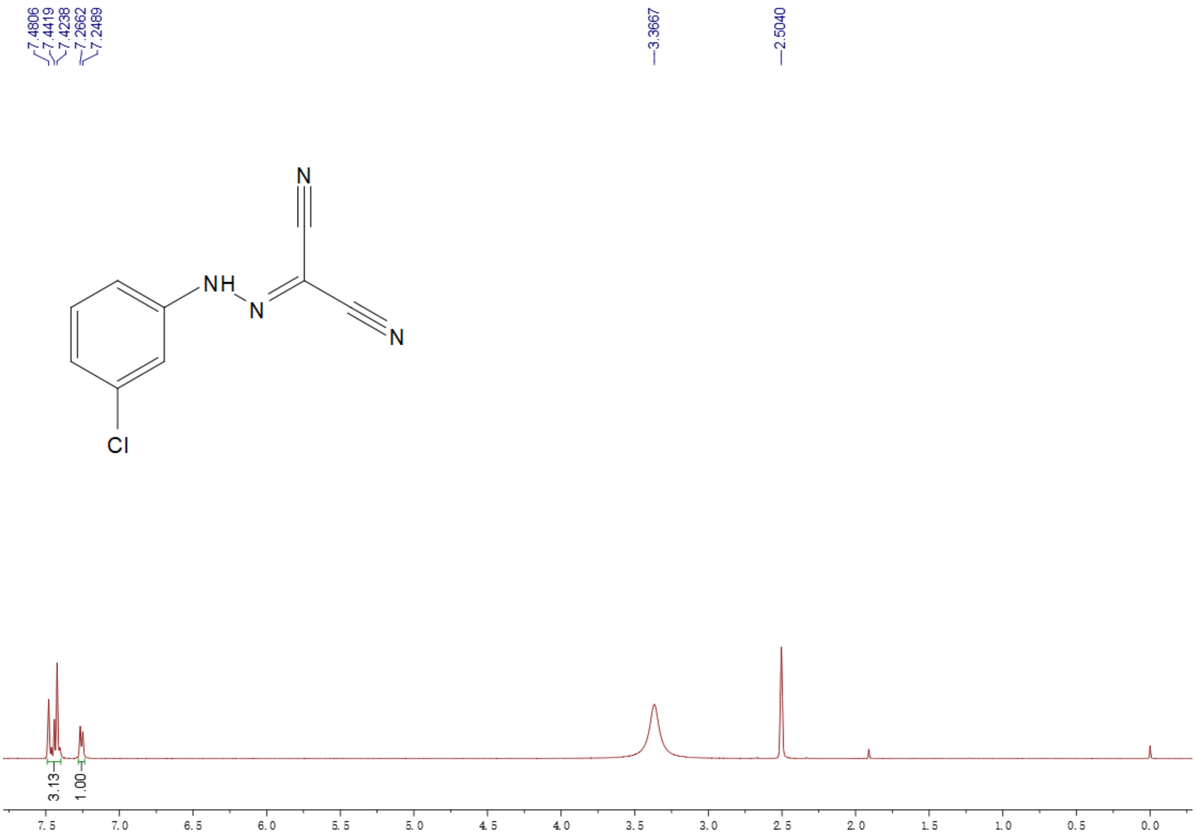

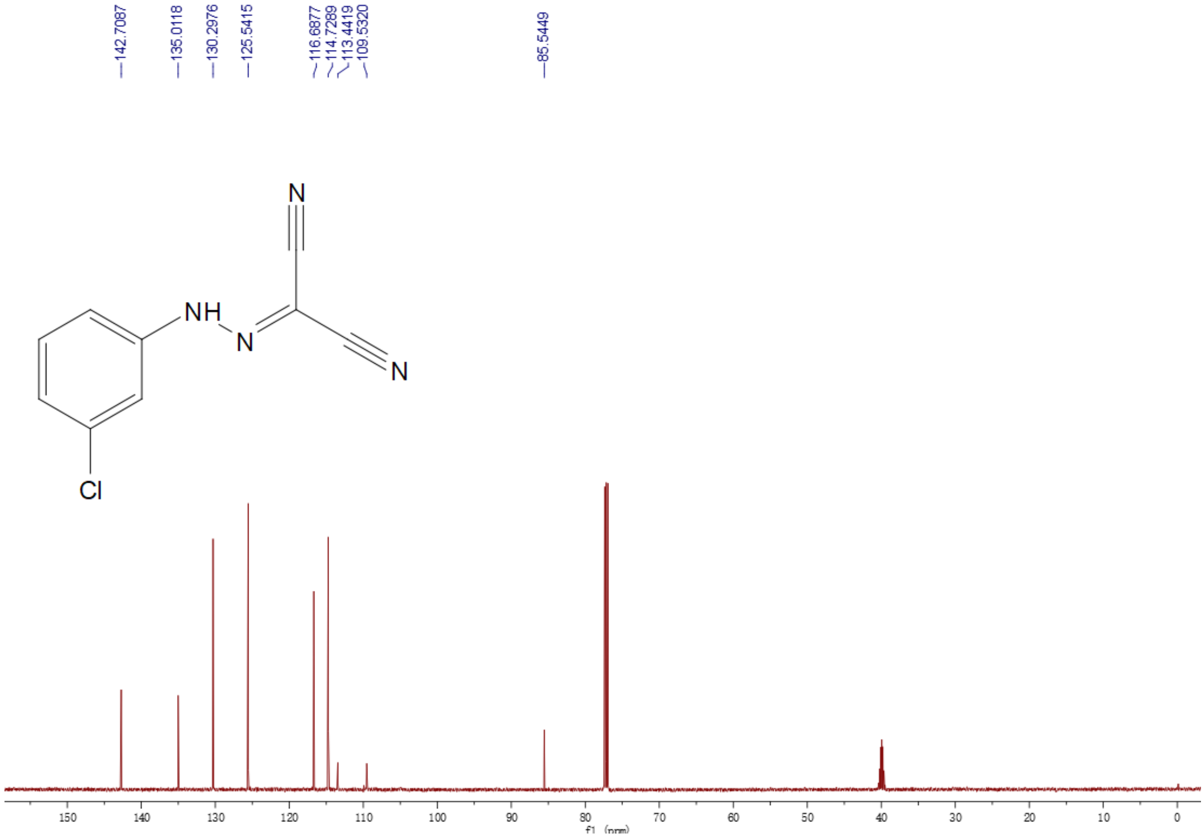

Figure S1. ^{1}H-NMR and ^{13}C-NMR spectra of compound **2a**.

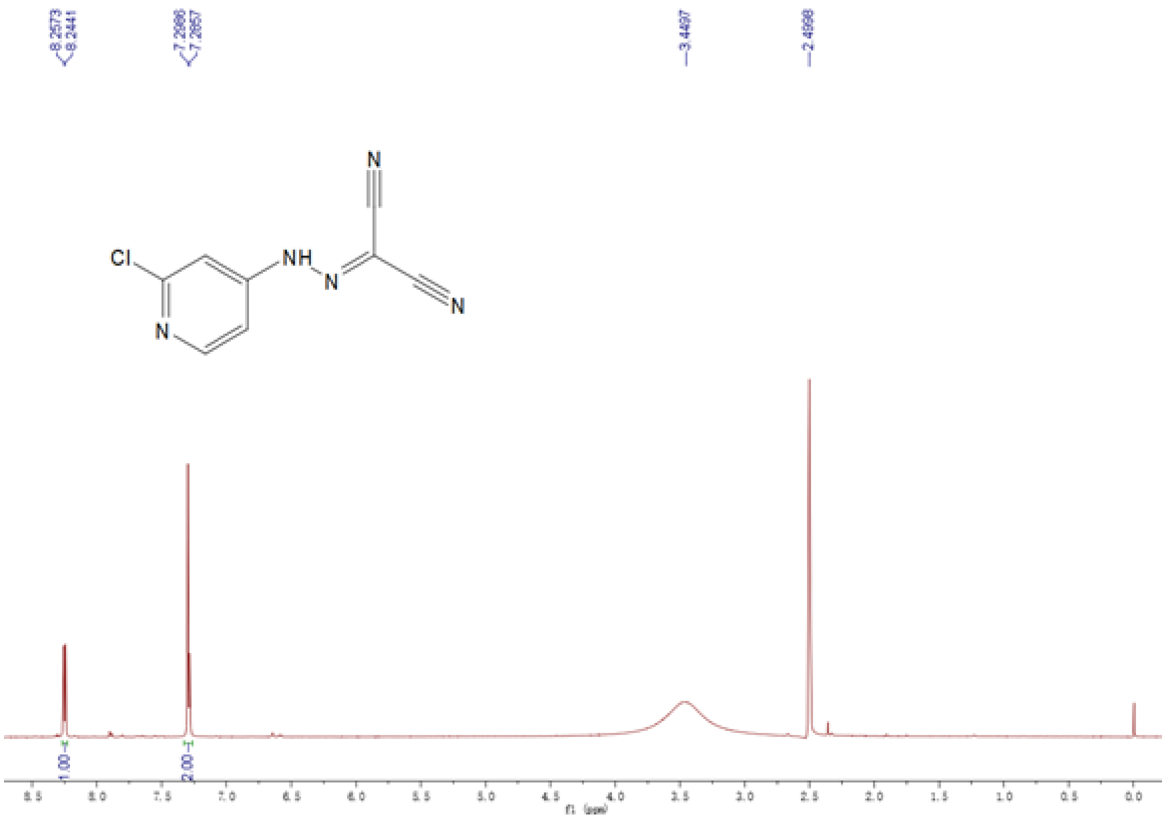

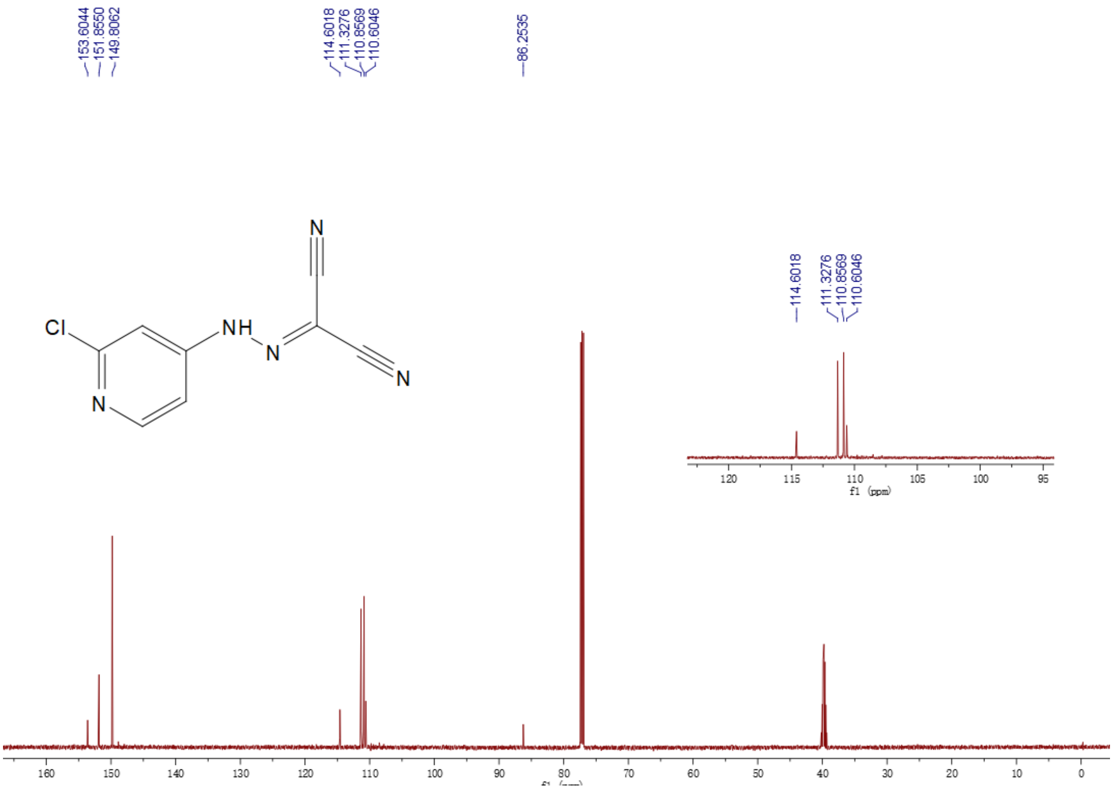

Figure S2. ^{1}H-NMR and ^{13}C-NMR spectra of compound **2b**.

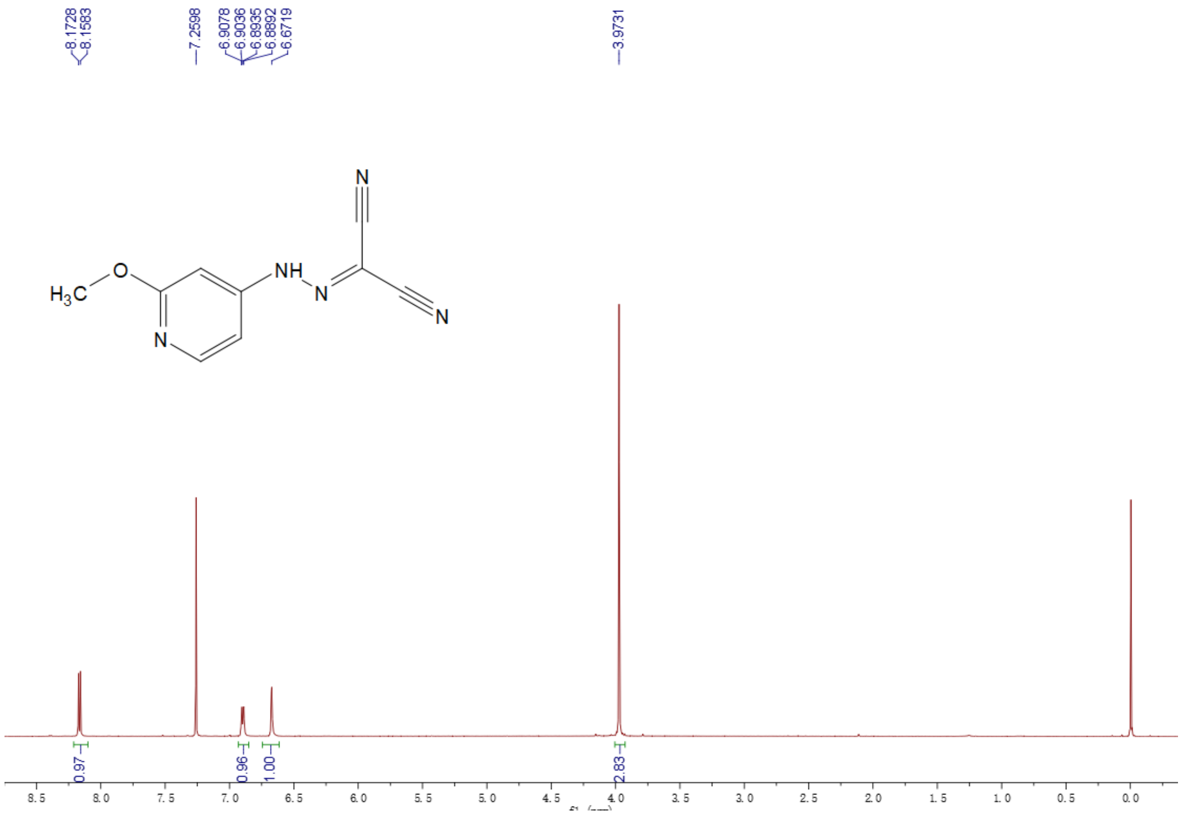

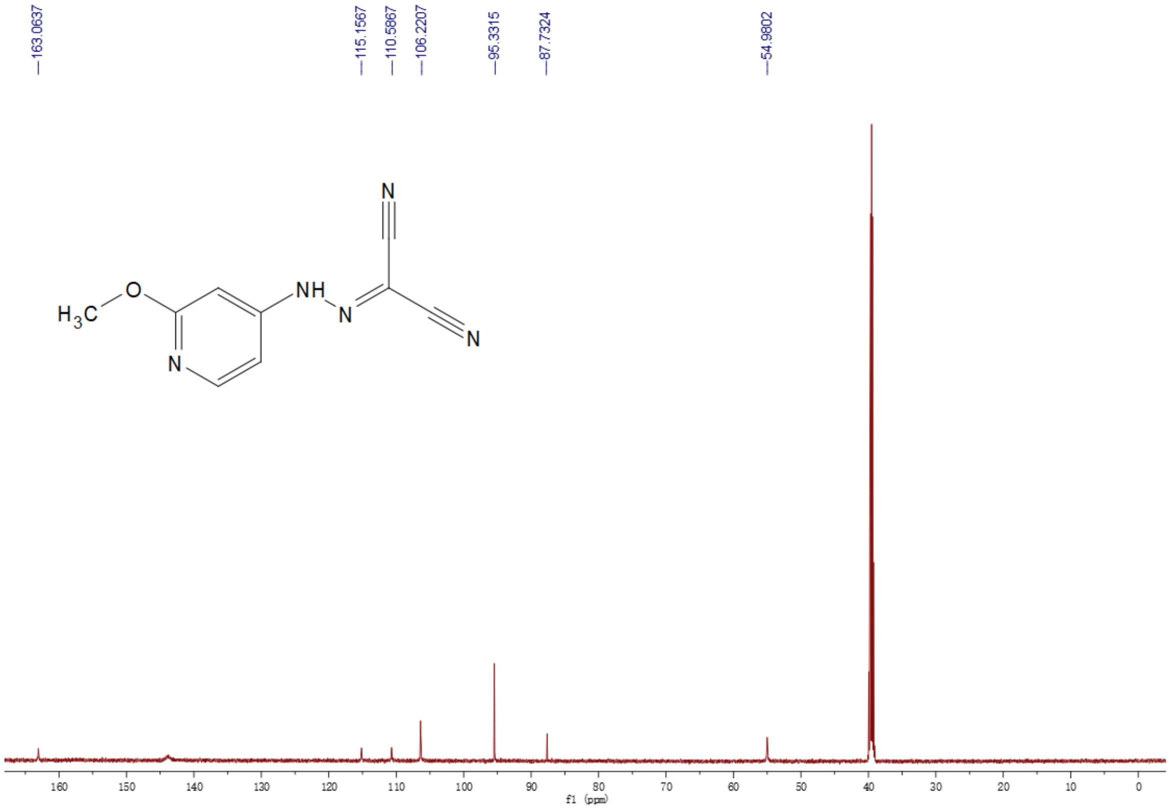

Figure S3. ^{1}H-NMR and ^{13}C-NMR spectra of compound **2c**.

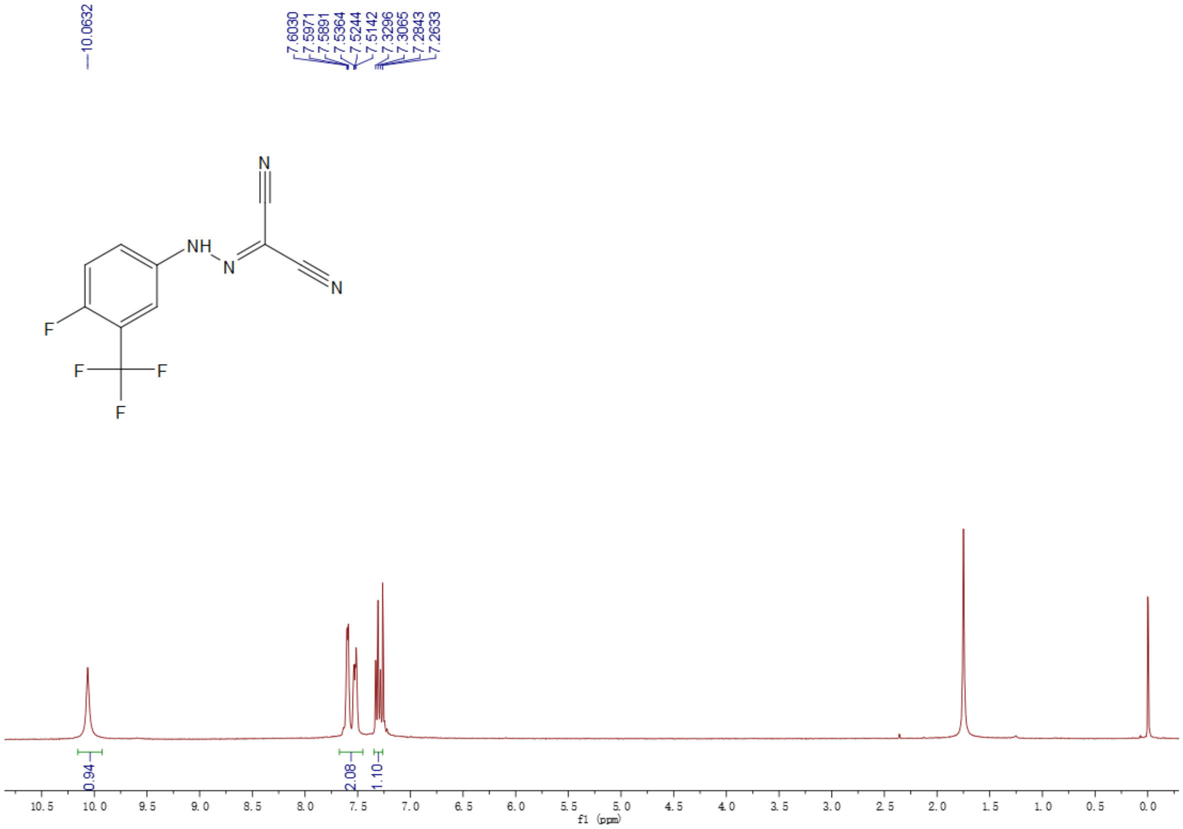

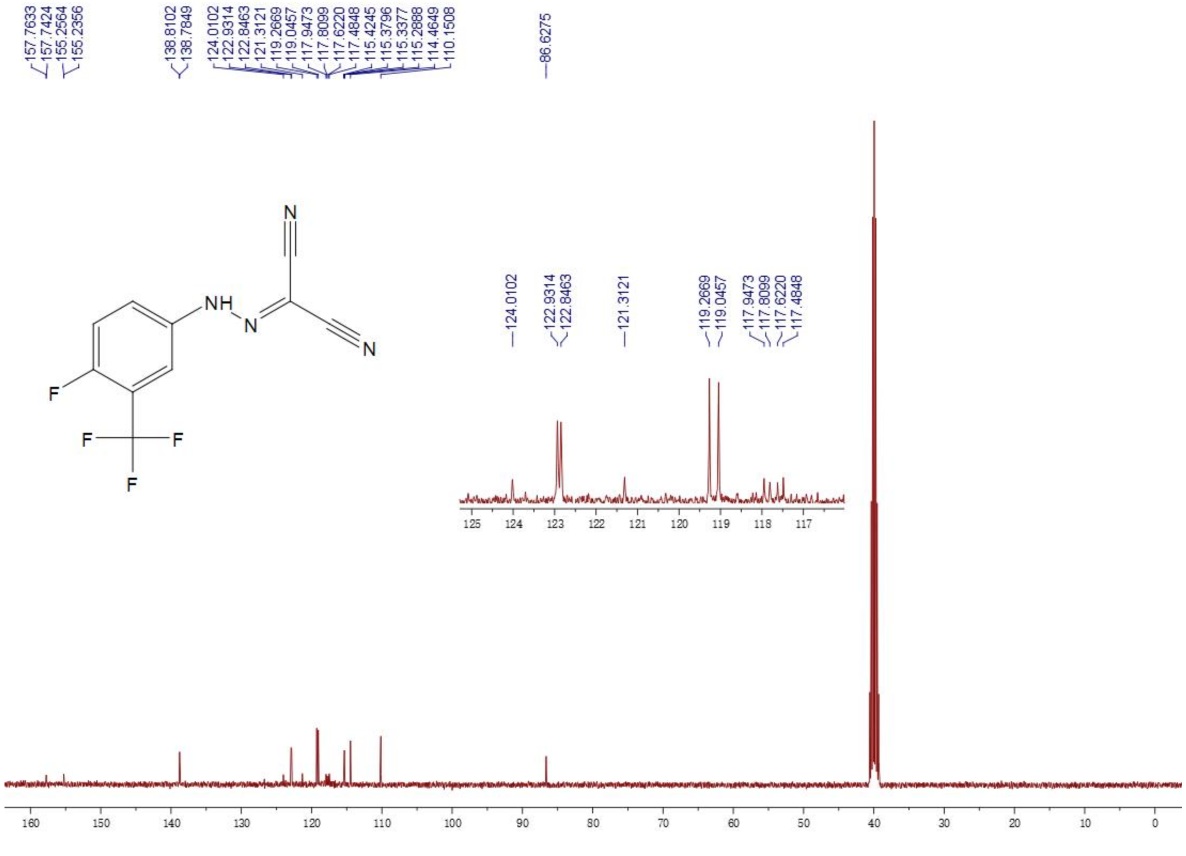

Figure S4. ^{1}H-NMR and ^{13}C-NMR spectra of compound **2d**.

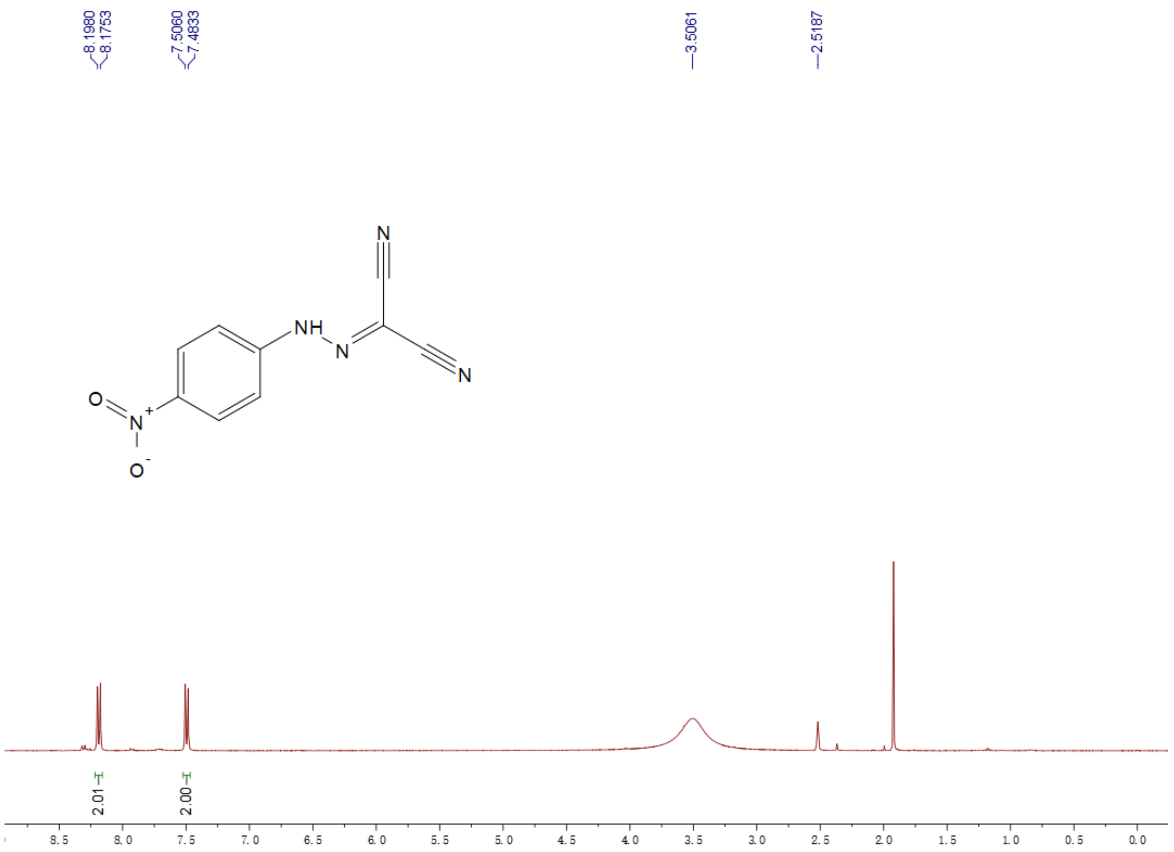

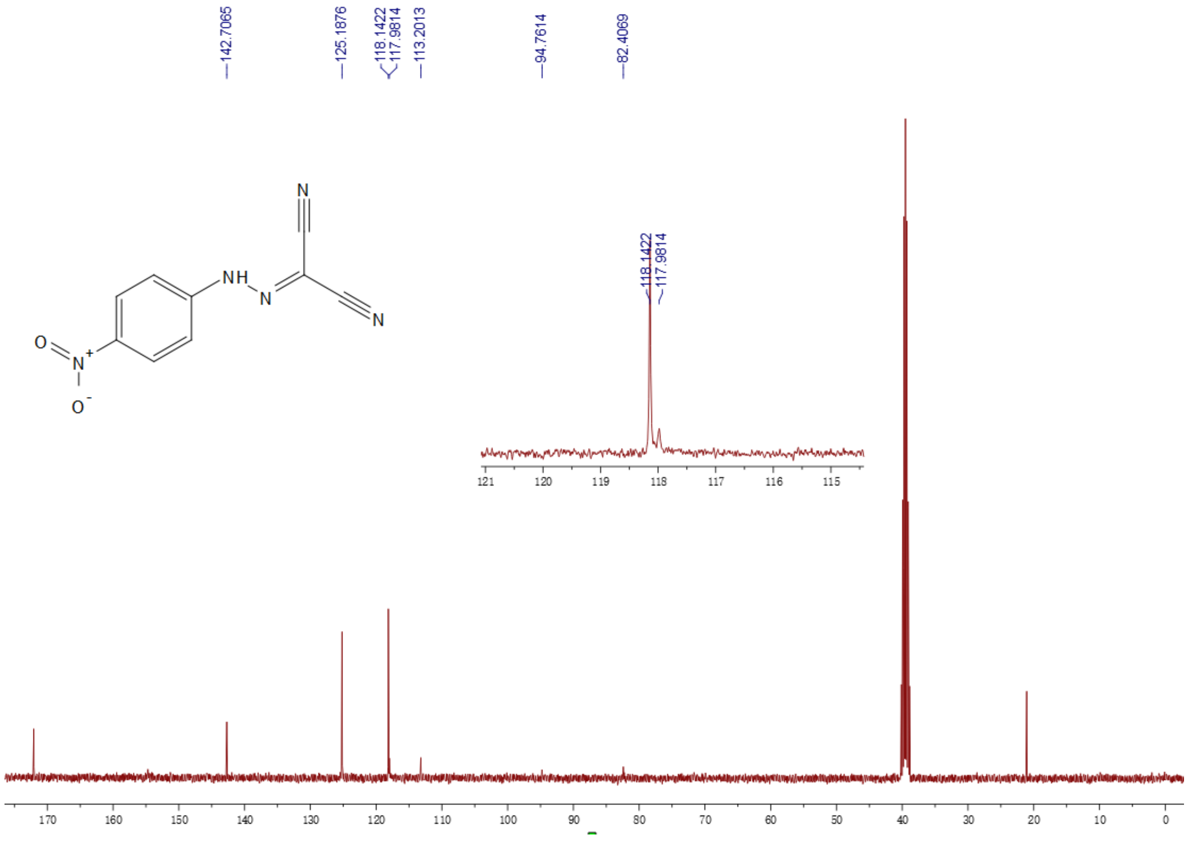

Figure S5. ¹H-NMR and ¹³C-NMR spectra of compound **2e**.

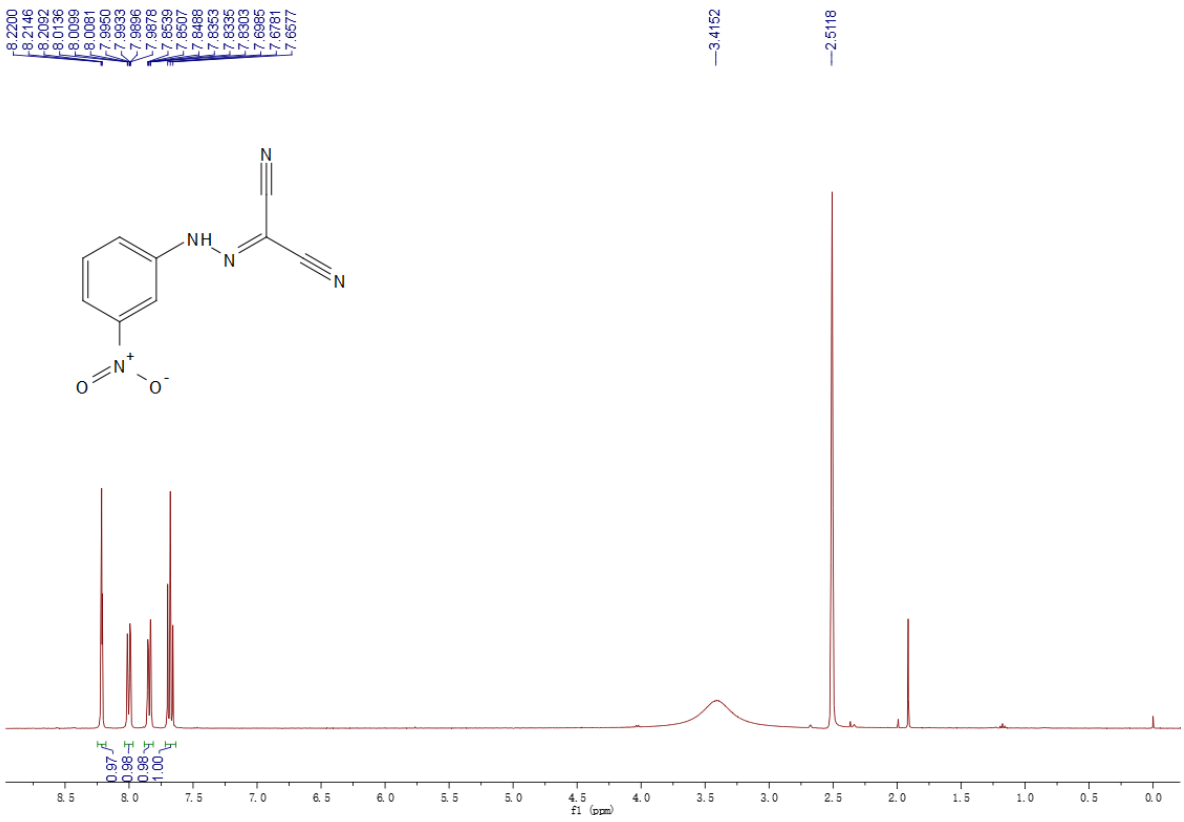

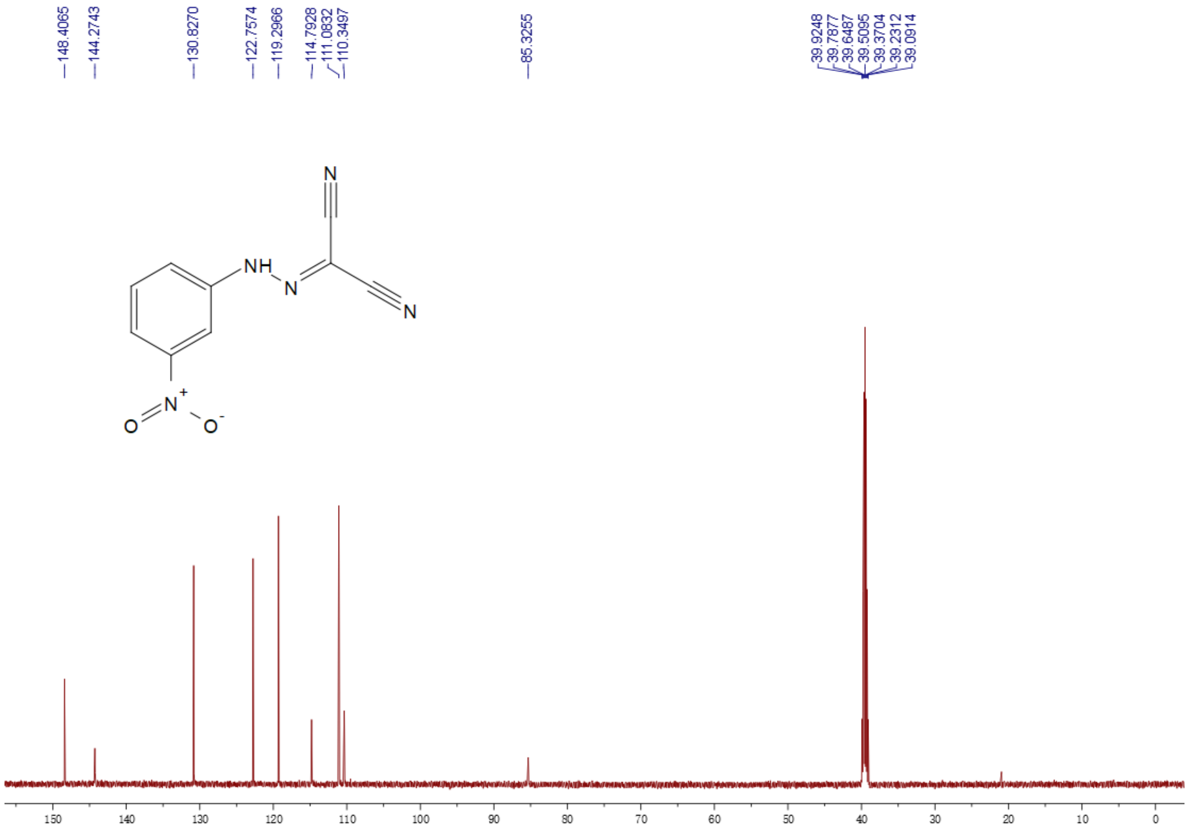

Figure S6. ^{1}H-NMR and ^{13}C-NMR spectra of compound **2f**.

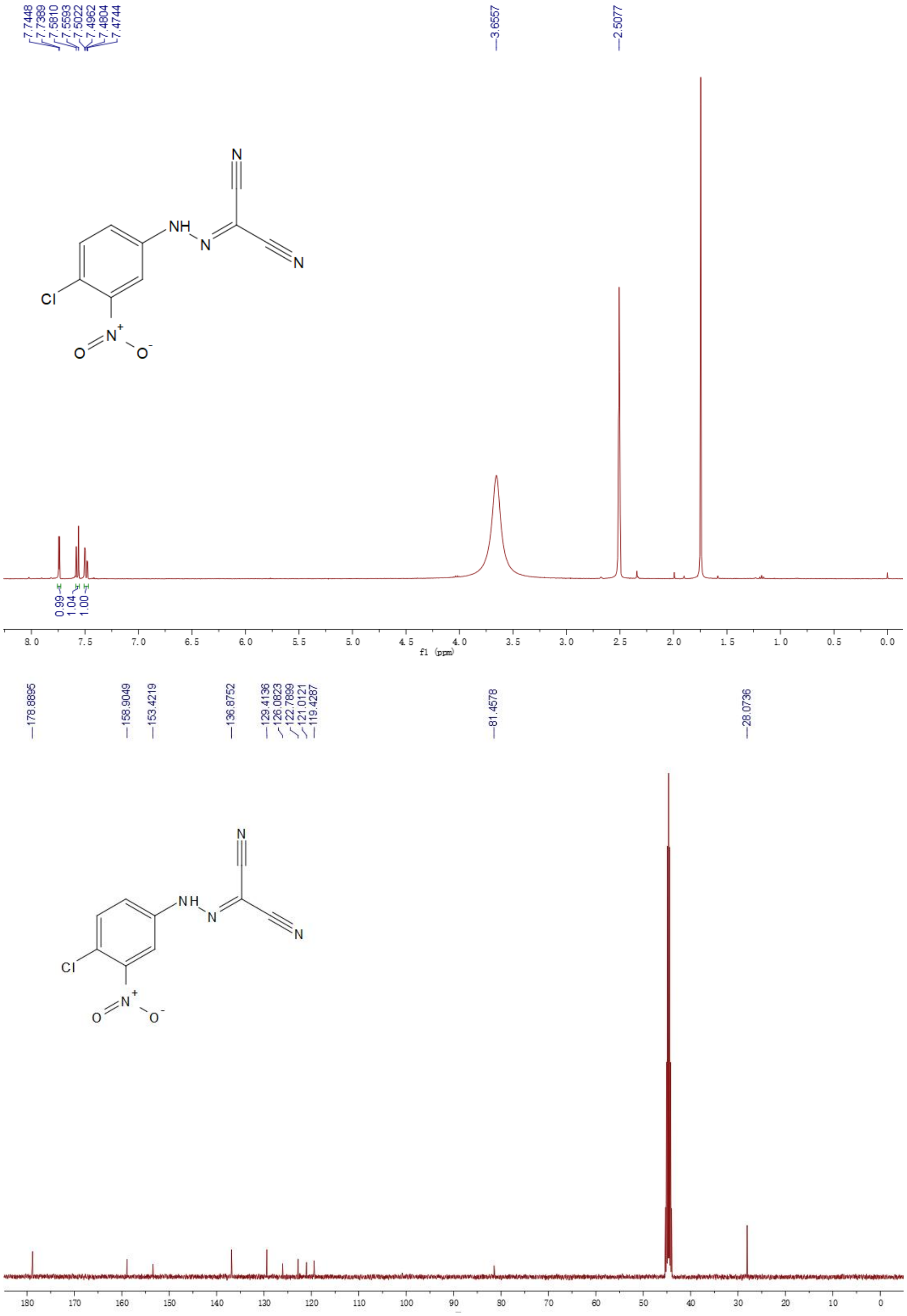

Figure S7. ^{1}H-NMR and ^{13}C-NMR spectra of compound **2g**

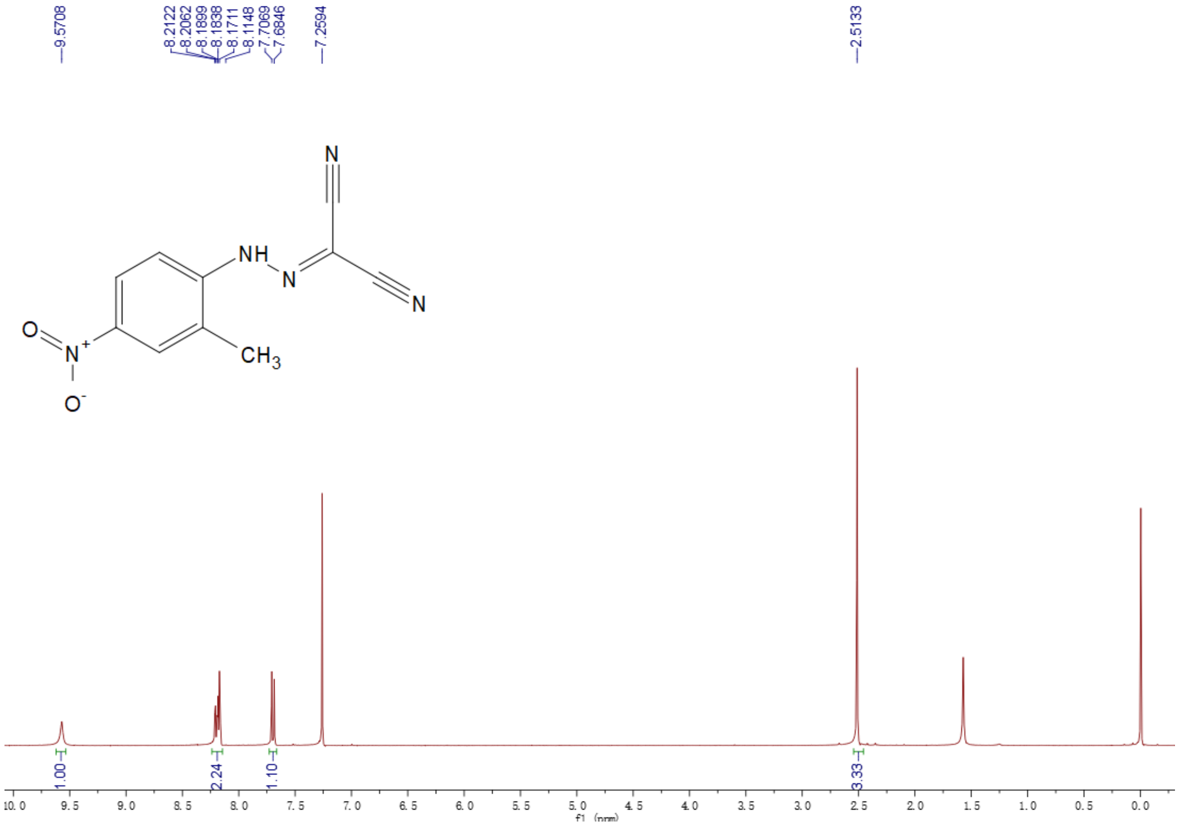

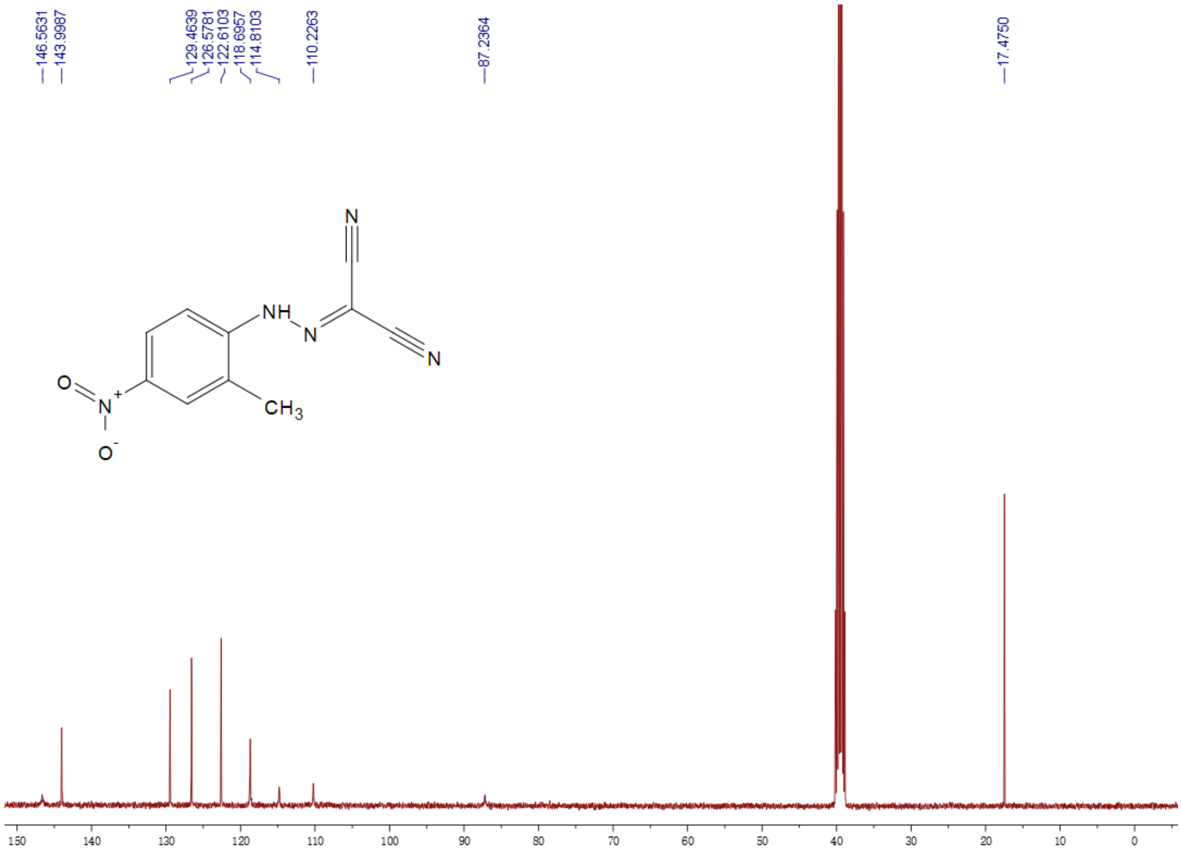

Figure S8. ^{1}H-NMR and ^{13}C-NMR spectra of compound **2h**

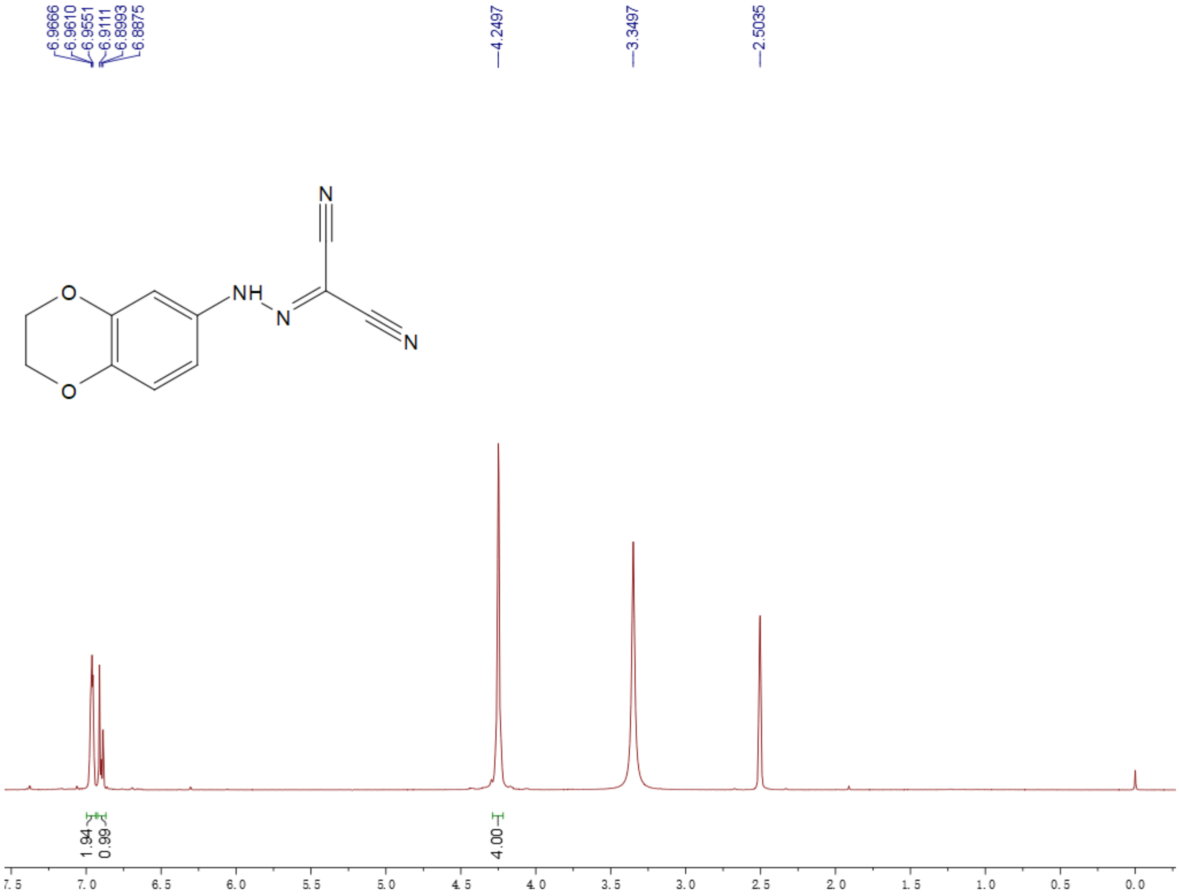

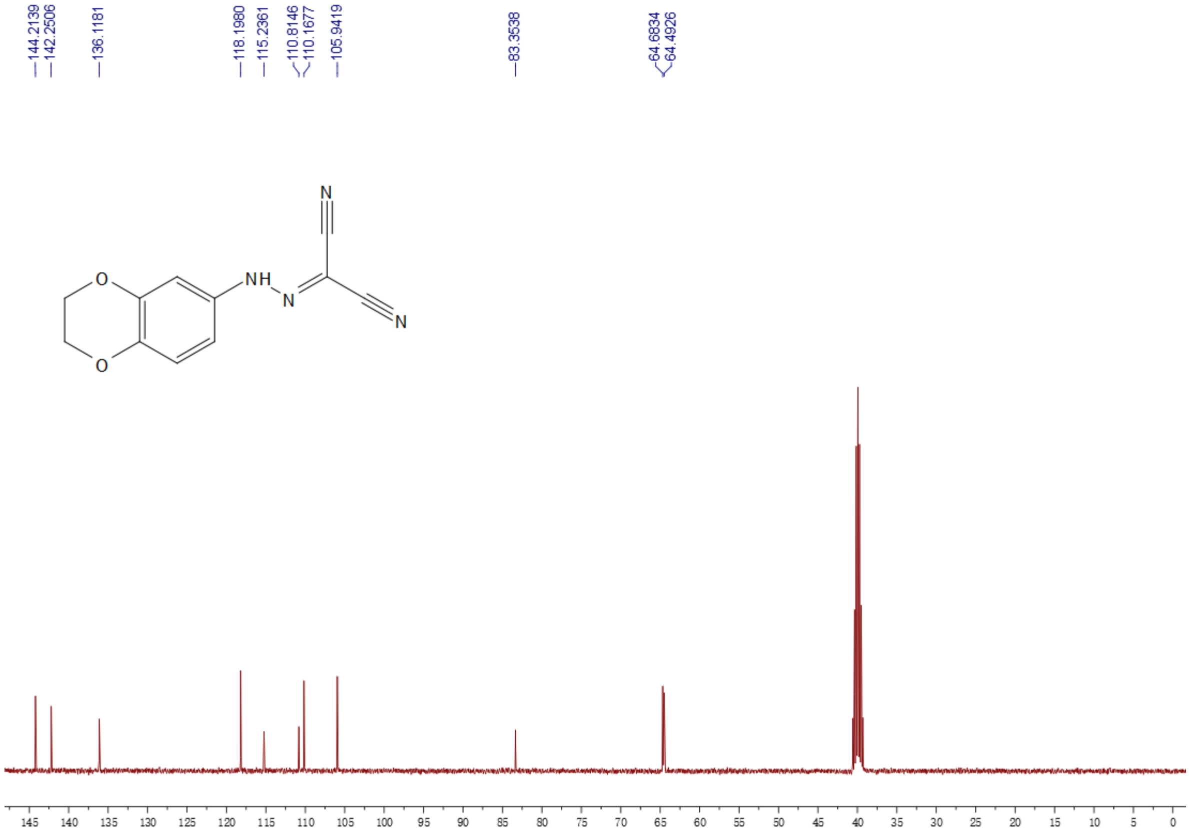

Figure S9. ^{1}H-NMR and ^{13}C-NMR spectra of compound **2i**.

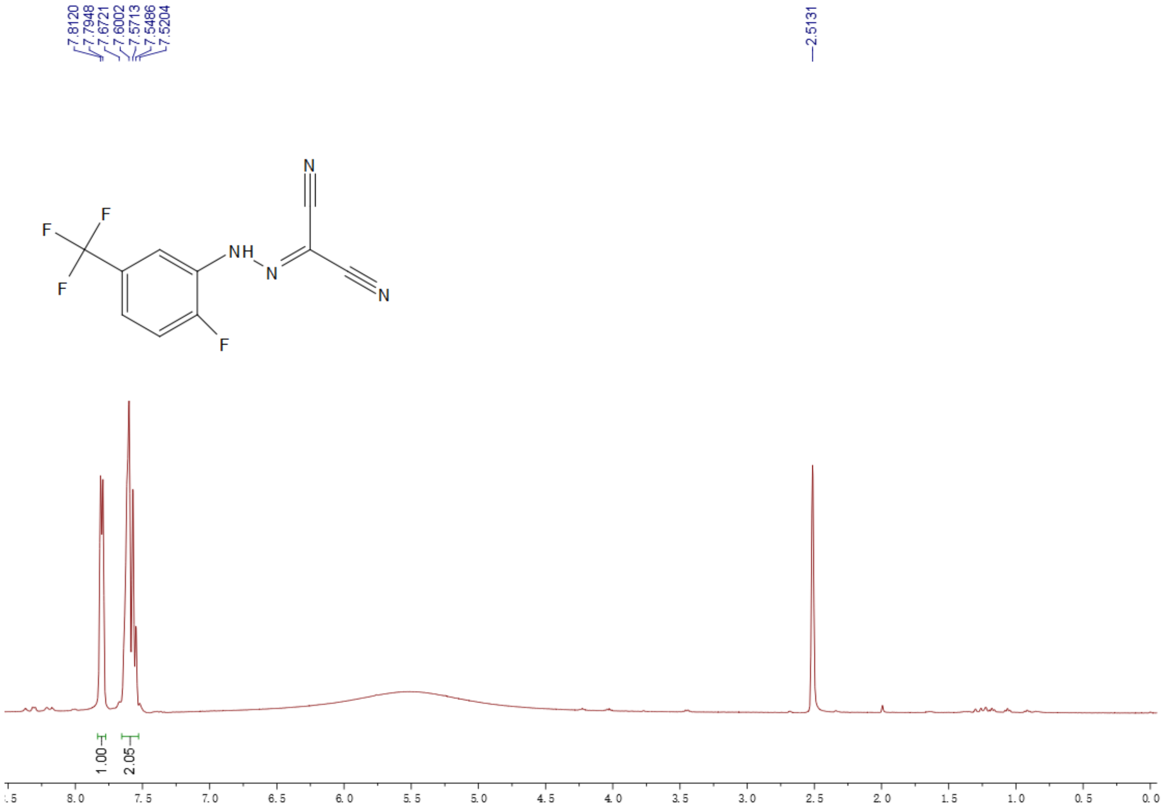

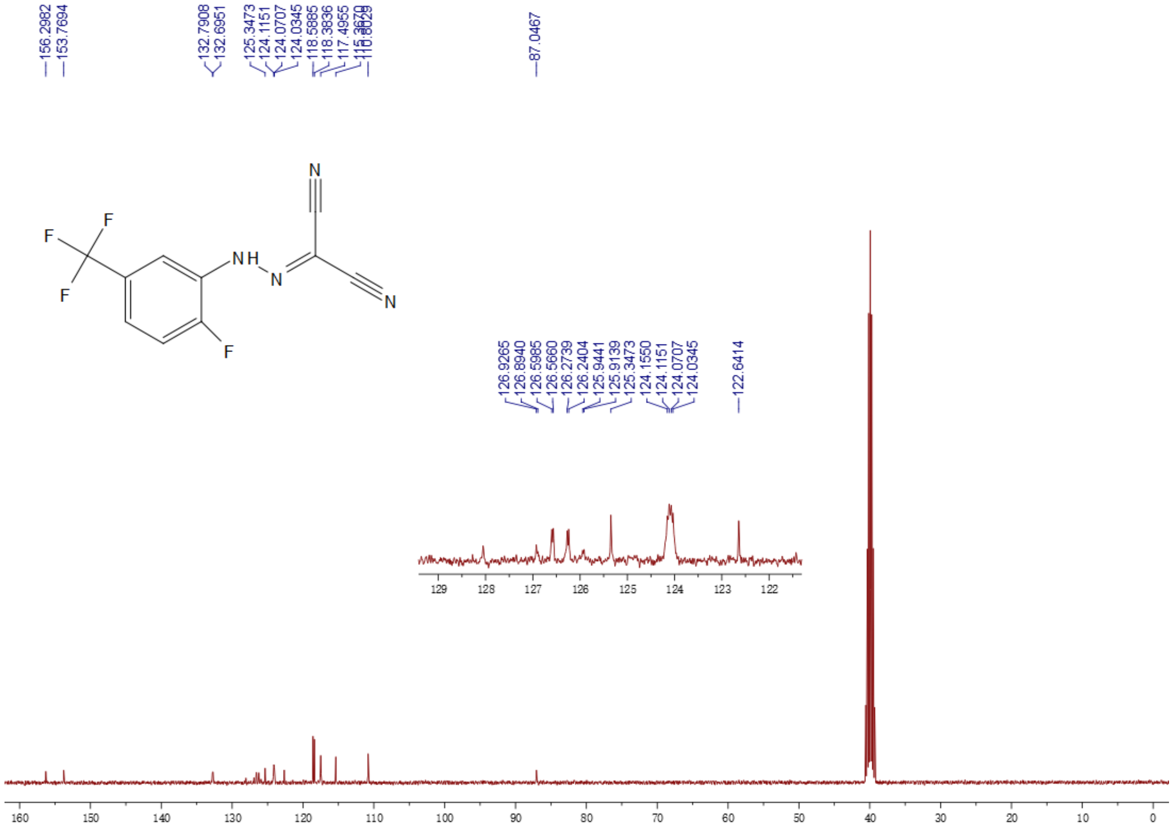

Figure S10. ^{1}H-NMR and ^{13}C-NMR spectra of compound **2j**.

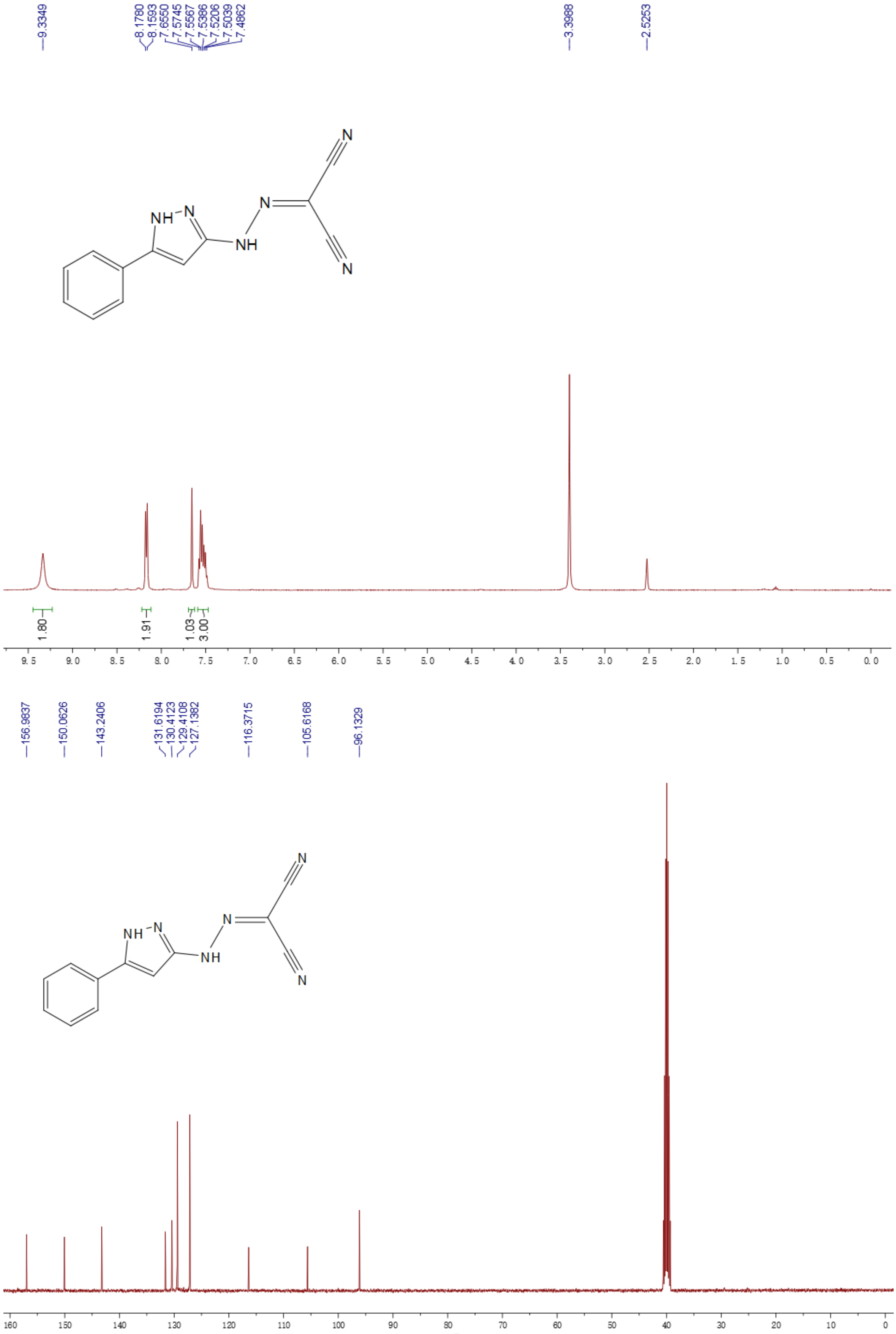

Figure S11. ^{1}H-NMR and ^{13}C-NMR spectra of compound **2k**.

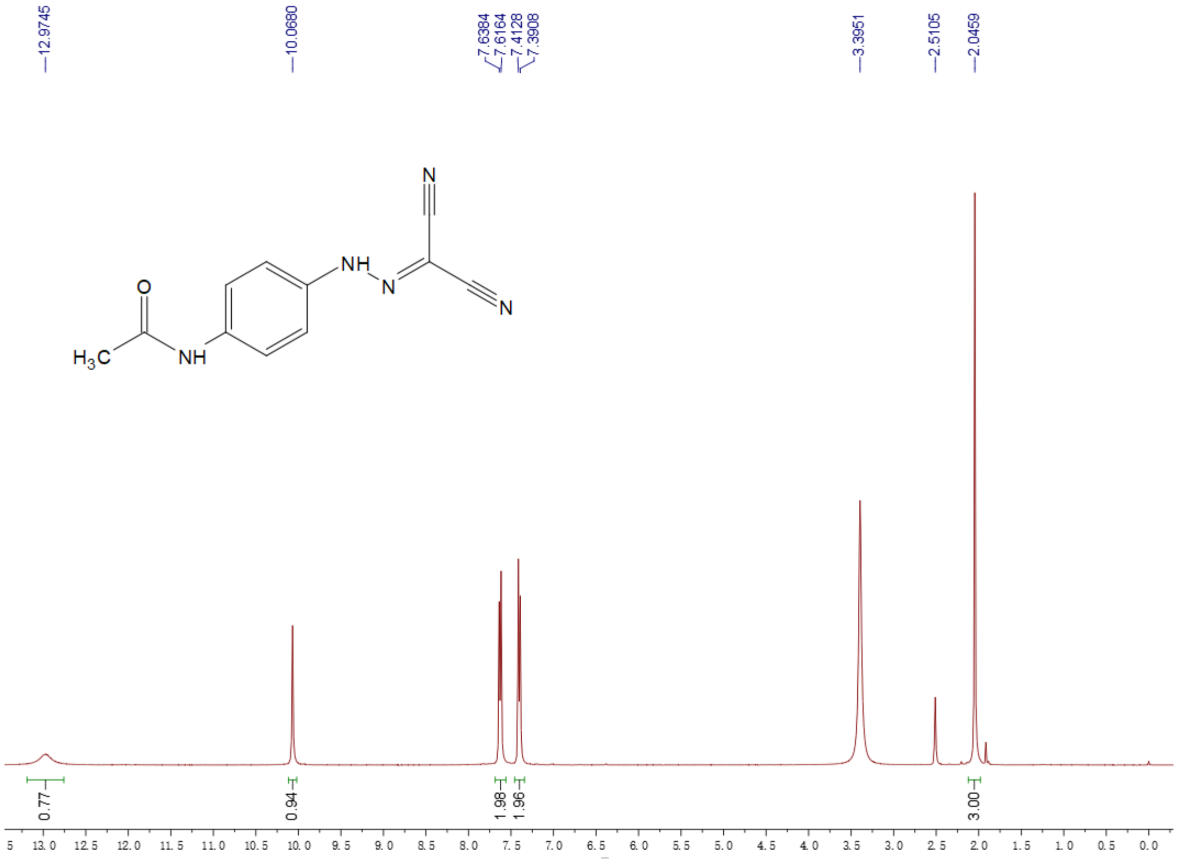

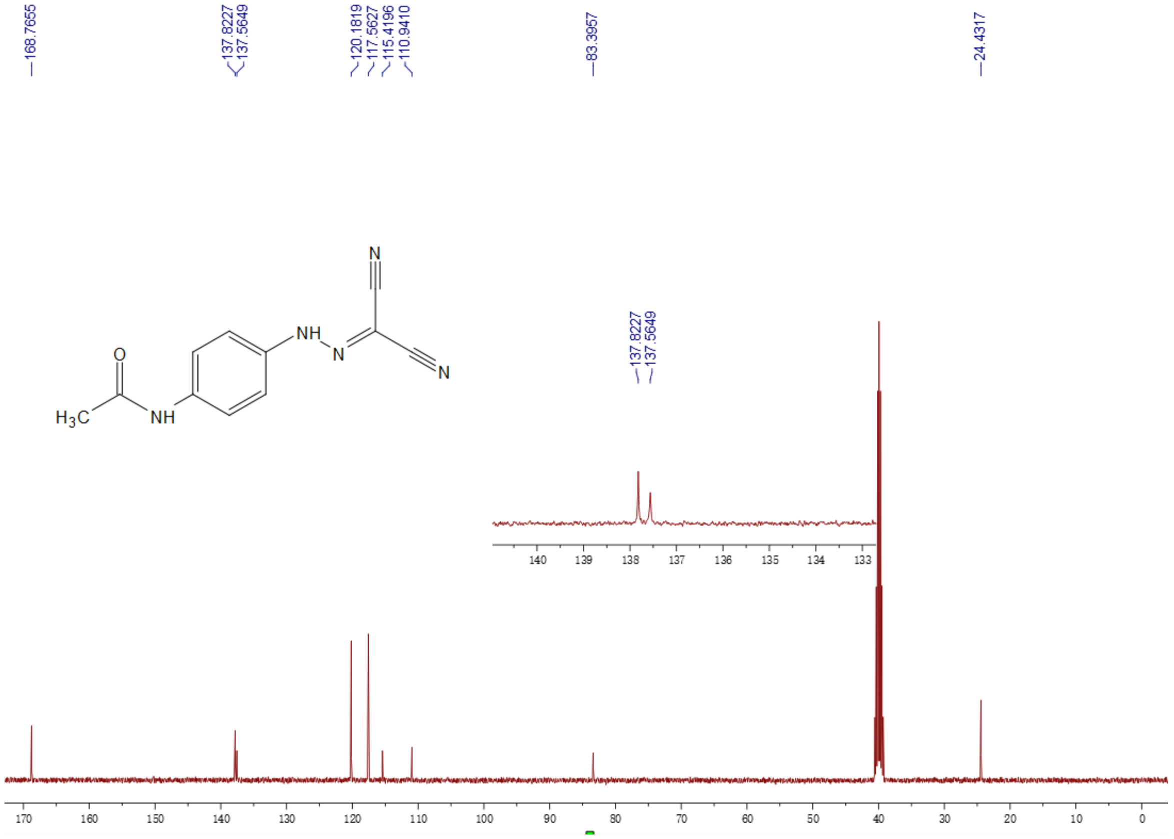

Figure S12. ^{1}H-NMR and ^{13}C-NMR spectra of compound **2l**.

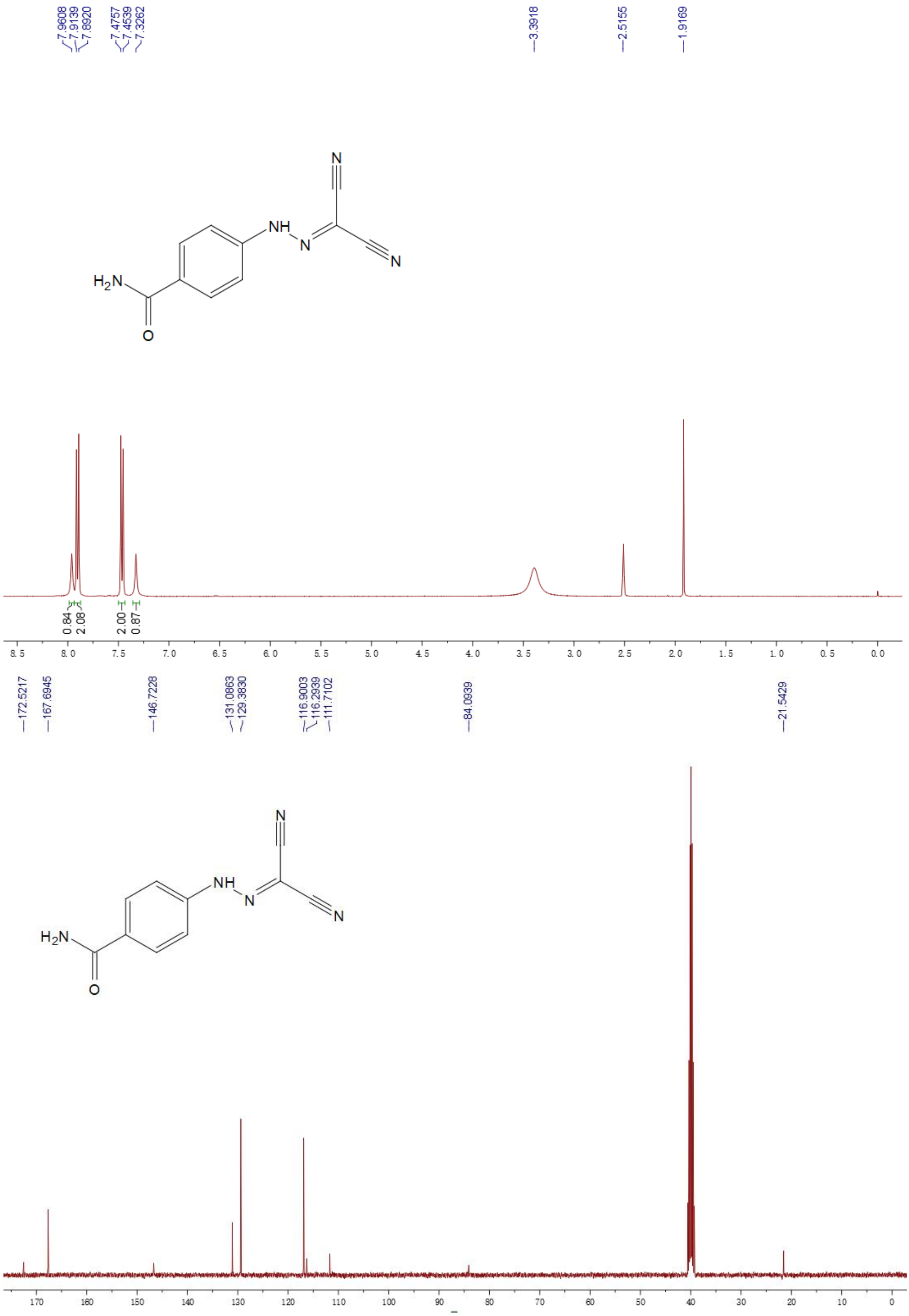

Figure S13. ^{1}H-NMR and ^{13}C-NMR spectra of compound **2m**.

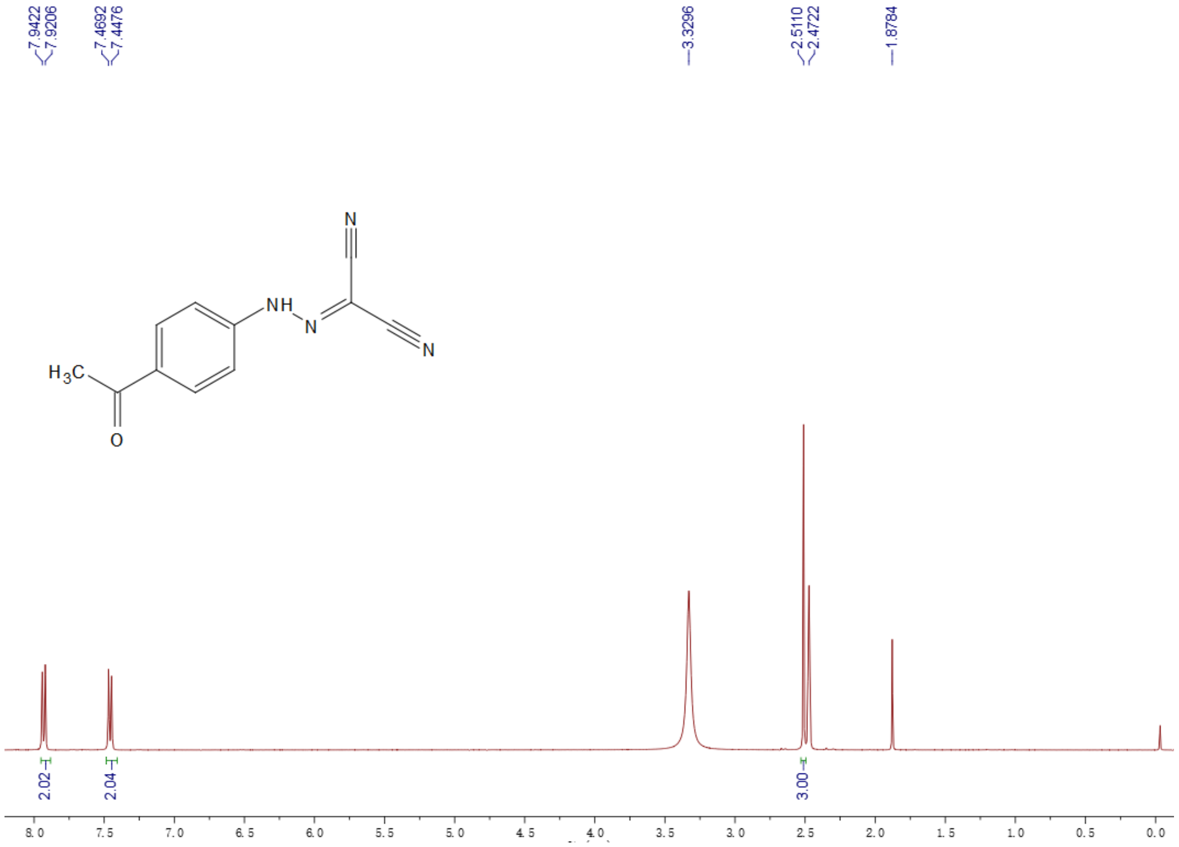

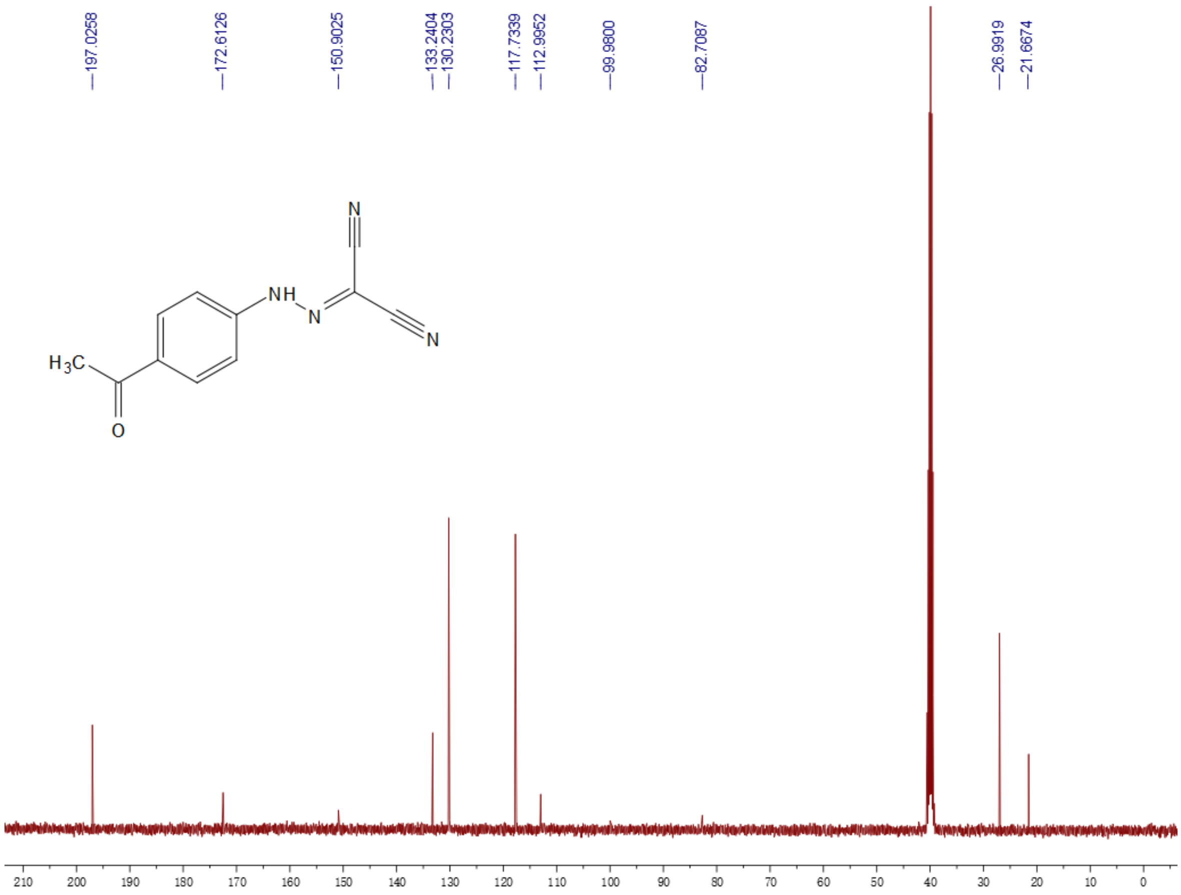

Figure S14. ^{1}H-NMR and ^{13}C-NMR spectra of compound **2n**.

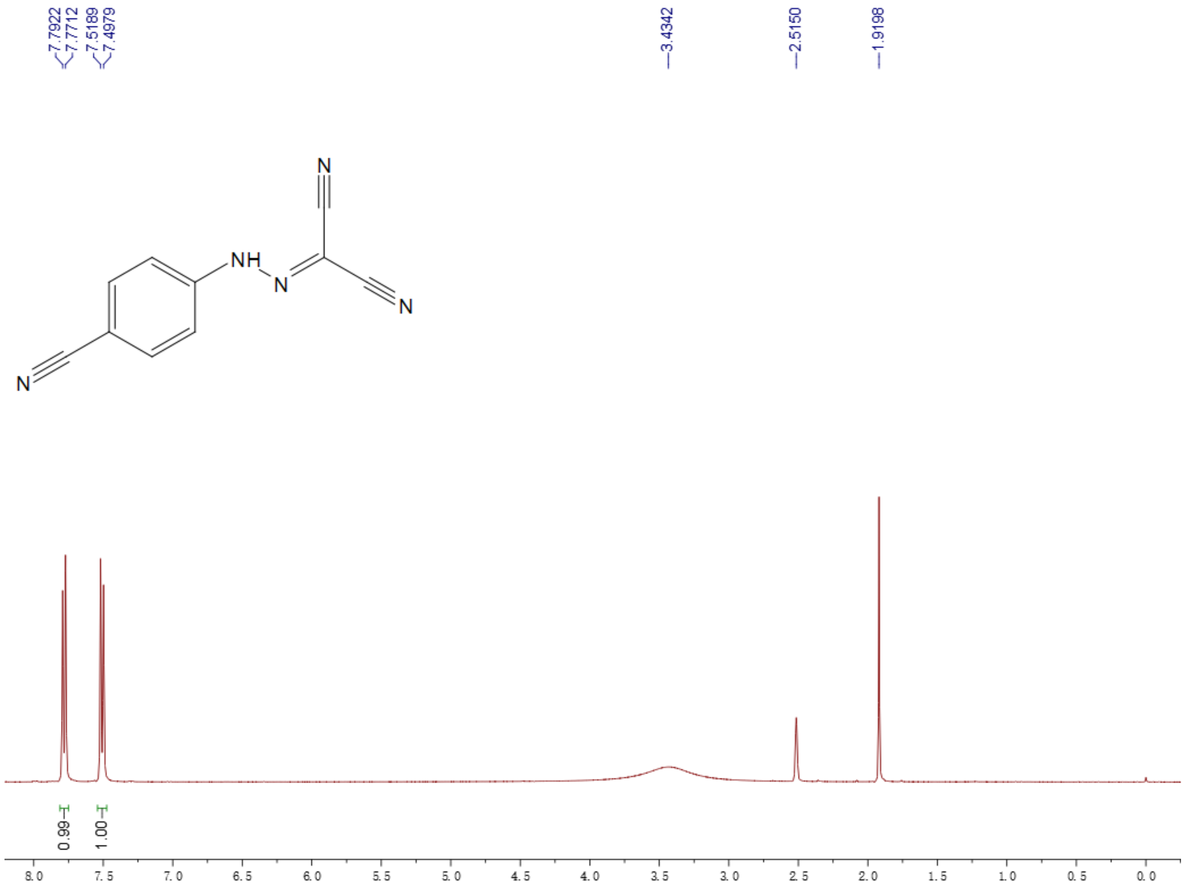

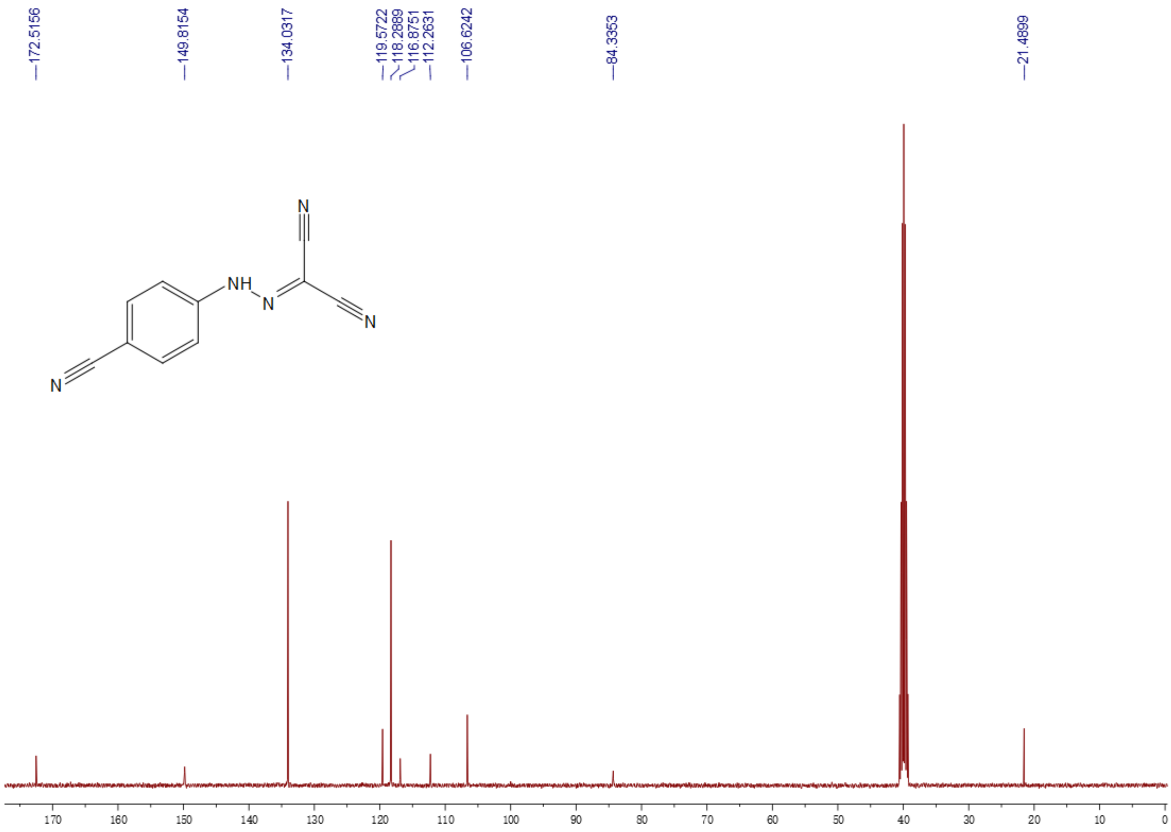

Figure S15. ^{1}H-NMR and ^{13}C-NMR spectra of compound **2o**.

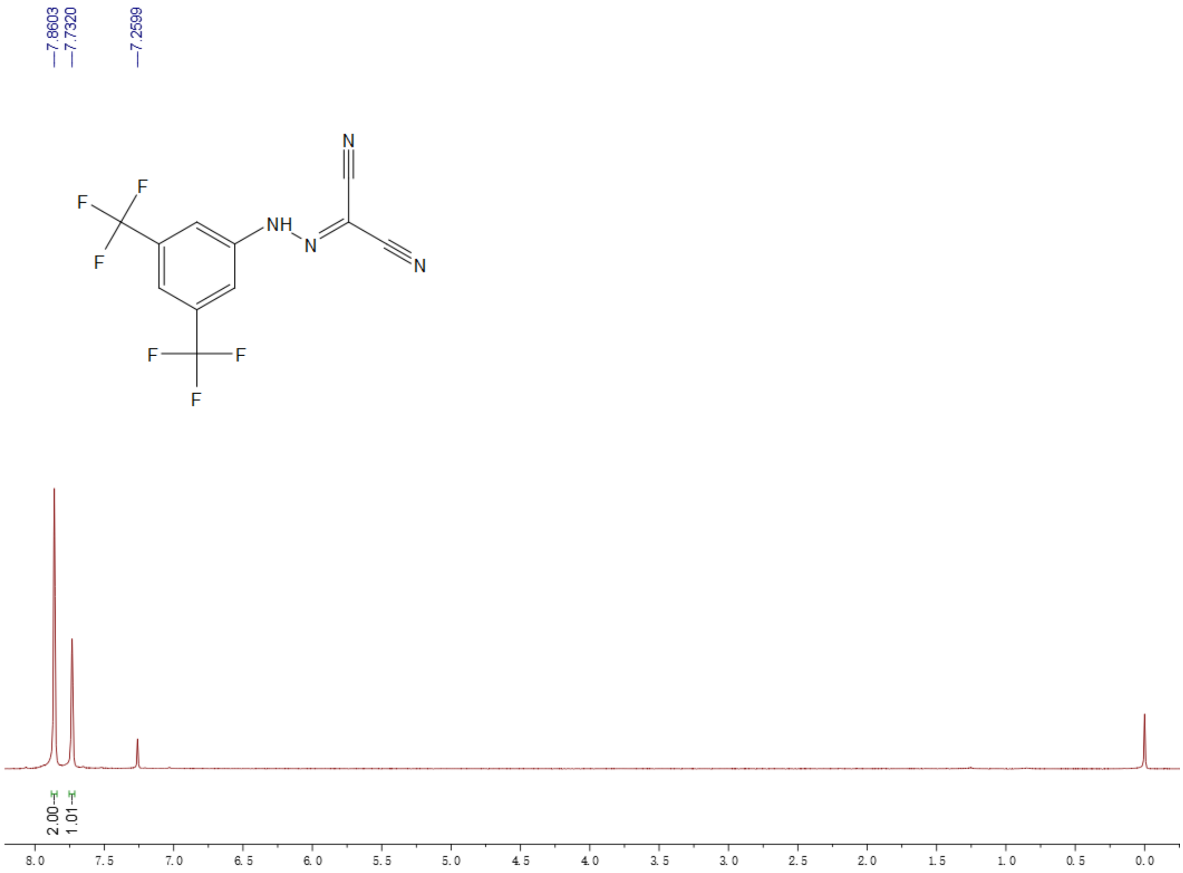

Figure S16. ^{1}H-NMR spectra of compound **2p**.

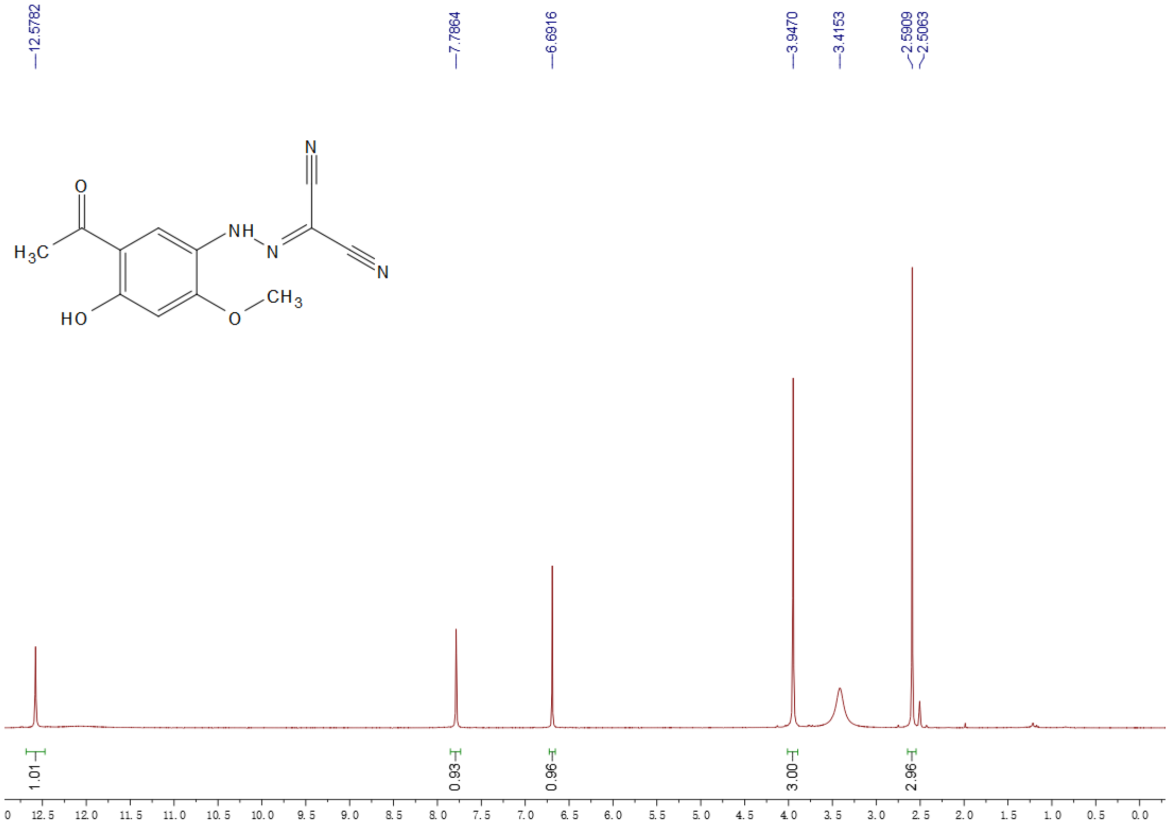

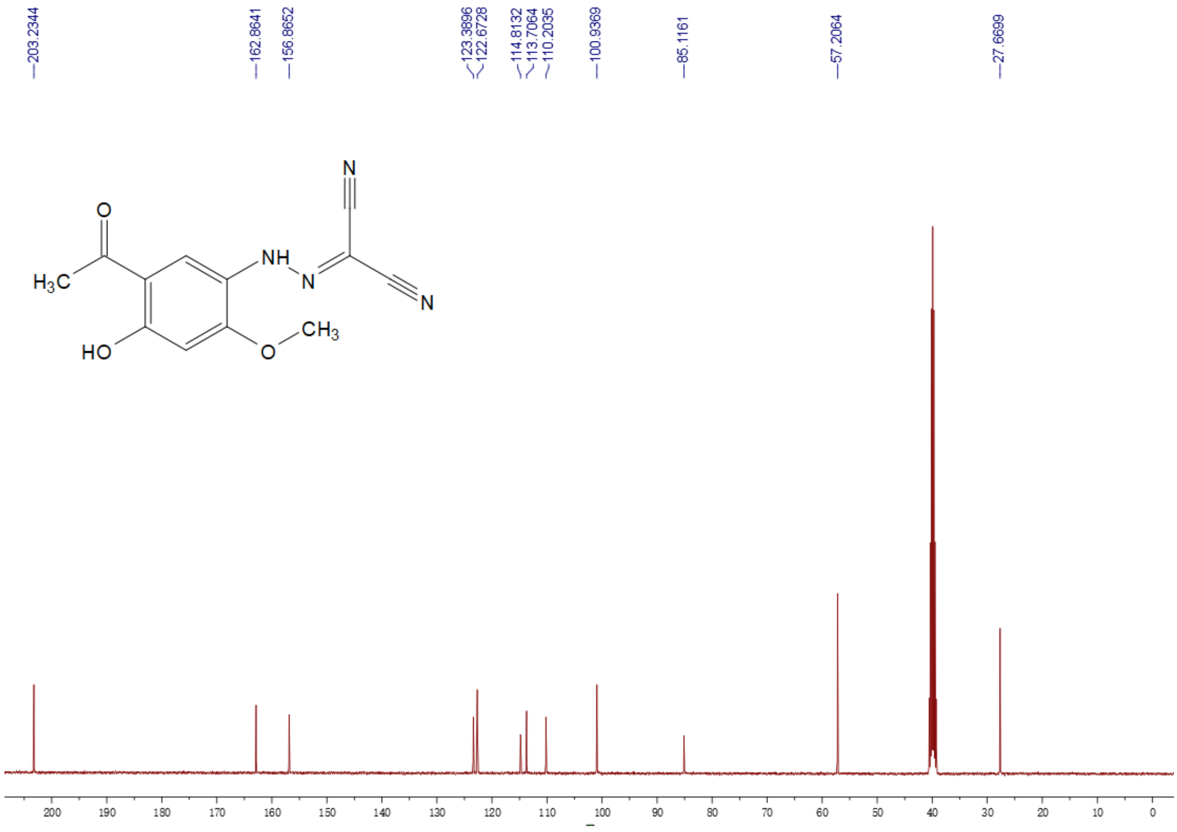

Figure S17. ^{1}H-NMR and ^{13}C-NMR spectra of compound **2q**.
